# Mitochondrial Transplantation Augments the Reparative Capacity of Macrophages Following Myocardial Injury

**DOI:** 10.1002/advs.202506337

**Published:** 2025-08-19

**Authors:** Yuning Zhang, Xiaolei Sun, Yawei Jin, Kanghui Chen, Lu Zhang, Xiong Gao, Mohan Li, Ze Yuan, Jianguo Jia, Aijun Sun, Junbo Ge

**Affiliations:** ^1^ Department of Cardiology Zhongshan Hospital Fudan University Shanghai Institute of Cardiovascular Diseases Shanghai 200032 China; ^2^ State Key Laboratory of Cardiovascular Diseases Zhongshan Hospital Fudan University Shanghai 200032 China; ^3^ NHC Key Laboratory of Ischemic Heart Diseases Shanghai 200032 China; ^4^ Key Laboratory of Viral Heart Diseases Chinese Academy of Medical Sciences Shanghai 200032 China; ^5^ National Clinical Research Center for Interventional Medicine Shanghai 200032 China; ^6^ Institutes of Biomedical Sciences Fudan University Shanghai 200032 China; ^7^ Qidong‐Fudan Innovative Institution of Medical Sciences Jiangsu 226200 China; ^8^ The Shanxi Medical University Taiyuan Shanxi 030001 China

**Keywords:** macrophage reparative capacity, mitochondrial transplantation, myocardial infarction

## Abstract

The pathologically remodeled myocardial ischemic microenvironment, characterized by sustained hypoxia, metabolic insufficiency, and accumulation of inflammatory mediators, severely disrupts mitochondrial homeostasis. This dysfunction establishes a self‐perpetuating cycle that impairs the coordinated healing cascade and compromises cardiac tissue repair following myocardial infarction (MI). To counteract these effects, a novel strategy of mitochondrial augmentation is proposed, whereby healthy exogenous mitochondria are introduced into macrophages to generate mitochondria‐transplanted macrophages (Mito‐T‐Macros or MTMs), which can resist post‐MI stress. Mitochondrial transplantation (MT) effectively induces macrophage polarization toward a reparative M2‐like phenotype, thereby enhancing pro‐healing functions, including migration, invasion, and phagocytosis. In vivo, MTM therapy enhances cardiac function after MI and attenuates left ventricular remodeling by reducing fibrosis, limiting apoptosis, and promoting angiogenesis. Mechanistically, MT accelerates the phenotypic transition of macrophages to a reparative state and prolongs their activity during the healing phase. Notably, a portion of the transplanted mitochondria are released from MTMs and subsequently internalized by cardiomyocytes, suggesting an additional mechanism of myocardial support. Overall, MT enhances the reparative capabilities of macrophages and contributes to the therapeutic efficacy of MTMs in ameliorating post‐MI cardiac remodeling. These findings support MTM therapy as a promising and innovative approach for repairing myocardial injury following MI.

## Graphical abstract

Mitochondrial transplantation induces macrophage polarization toward an anti‐inflammatory M2 phenotype, enhances their reparative capacities, and facilitates mitochondrial transfer to cardiomyocytes, collectively promoting tissue repair and functional recovery post‐myocardial infarction. Tumor necrosis factor‐alpha (TNF‐α), Interleukin‐1 beta (IL‐1β), Cluster of Differentiation 206 (CD206), Thrombospondin‐1 (TSP‐1), Mer Tyrosine Kinase (MerTK), Transforming Growth Factor‐beta 1 (TGF‐β1), Interleukin‐10 (IL‐10), Vascular Endothelial Growth Factor‐alpha (VEGF‐α).

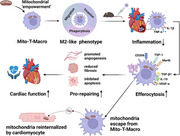

## 1 Introduction

Cardiovascular disease remains one of the most significant global health burdens, with ischemic heart disease (IHD) being the leading cause of death among affected patients.^[^ ^1^ ^]^ The immune response plays a pivotal role in the progression of myocardial infarction (MI), critically influencing disease outcomes.^[^ ^2^ ^]^ Among immune cells, macrophages exhibit remarkable chemotactic and plastic properties, allowing them to rapidly respond to stress signals. Through phenotypic transformation, they precisely modulate disease processes and remain one of the most active cell types at every stage following MI.^[^ ^3^, ^4^ ^]^

The post‐MI ischemic microenvironment is characterized by hypoxia, energy crisis, oxidative stress, inflammatory responses, and extracellular matrix remodeling, all of which collectively impair macrophage function within the injured myocardium. Hypoxia leads to ATP depletion and the excessive production of reactive oxygen species (ROS), simultaneously triggering the abnormal opening of mitochondrial permeability transition pores (mPTPs), which results in the dissipation of membrane potential and calcium overload.^[^ ^5^, ^6^ ^]^ These factors collectively contribute to mitochondrial dysfunction in macrophages, hindering their transition to a reparative phenotype characterized by oxidative phosphorylation (OXPHOS) metabolism.^[^ ^7^, ^8^, ^9^ ^]^ Therefore, enhancing macrophage repair functions through mitochondria‐targeted strategies is a promising approach for promoting myocardial recovery after MI.

Current interventions, such as mitochondria‐targeted ROS clearance and inhibition of mitochondrial DNA (mtDNA) oxidation, have shown promise in partially rescuing mitochondrial dysfunction in macrophages.^[^ ^8^, ^10^ ^]^ However, mitochondrial damage within the ischemic microenvironment is often extensive, and these interventions are generally limited to single targets, proving largely ineffective when macrophages contain already dysfunctional mitochondria or damaged mtDNA.

The concept of “engineered macrophages” has emerged as an encouraging therapeutic strategy.^[^ ^11^ ^]^ One notable approach involves the development of chimeric antigen receptor macrophages (CAR‐Ms), which are engineered to specifically bind to target antigens, thereby enhancing their phagocytic activity against target cells.^[^ ^12^, ^13^ ^]^ Additionally, nanotechnology has been utilized to load therapeutic agents onto macrophages, transforming them into potential drug‐delivery vehicles.^[^ ^14^ ^]^ These examples demonstrate that macrophages can be engineered and programmed to dynamically execute complex biological functions while retaining their essential characteristics as living cells.

A novel and complementary approach involves mitochondrial transplantation (MT) for the repair of myocardial injury. In 2017, McCully et al.^[^ ^15^ ^]^ have pioneered clinical trials to investigate MT as a therapeutic strategy for myocardial ischemia‐reperfusion injury (MIRI). Our research has also demonstrated the safety and efficacy of targeted MT for IHD.^[^ ^16^ ^]^

Building on this foundation, we propose an innovative strategy to engineer macrophages by preparing mitochondria‐transplanted macrophages (Mito‐T‐Macros or MTMs). This approach aims to leverage MTMs as a novel form of engineered macrophages for treating myocardial injury following MI. By integrating mitochondrial repair mechanisms with the intrinsic plasticity and functionality of macrophages, this strategy holds the potential for promoting myocardial recovery in a targeted and efficient manner.

This study has been designed to utilize MT to enhance the reparative capabilities of macrophages. We have discovered that MT facilitates the transformation of macrophages into the M2 phenotype in vitro, substantially augmenting their reparative functions, including enhanced migration, invasion, and phagocytosis. In vivo, MTMs enhance cardiac function by promoting the early differentiation and sustained infiltration of reparative CD206^+^ macrophages, resulting in reduced fibrosis and apoptosis as well as enhanced angiogenesis. Our findings indicate that mitochondria‐based macrophage augmentation holds substantial potential as a novel therapeutic strategy for the clinical management of MI.

## 2 Results

### 2.1 MT Promotes the M2‐Like Macrophage Phenotype

**Figure 1:**
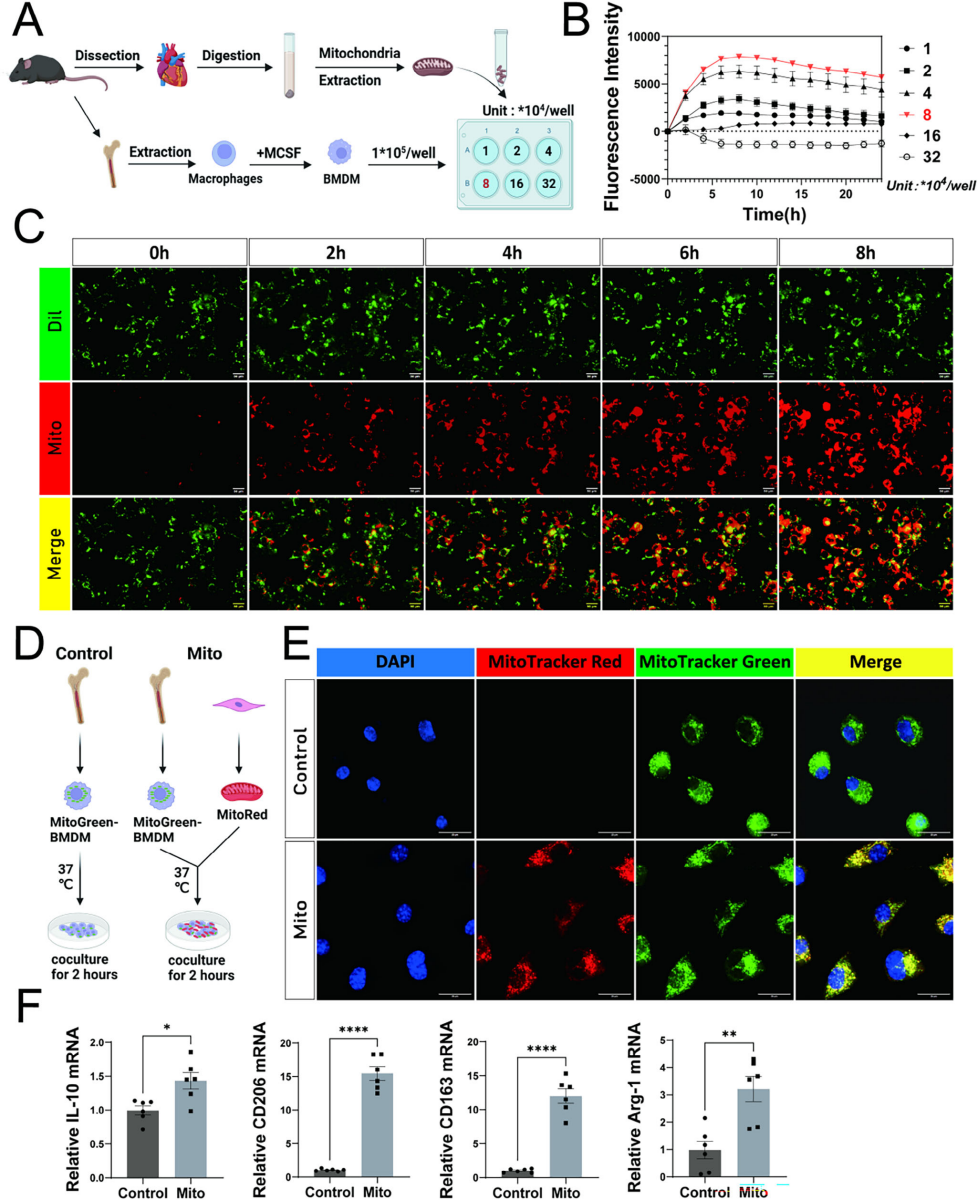
MT promotes the M2‐like macrophage phenotype. A) Schematic diagram illustrating the experimental workflow for co‐culturing BMDMs (1*10^5^) with mitochondrial suspension of different content (1*10^4^, 2*10^4^, 4*10^4^, 8*10^4^, 16*10^4^, 32*10^4^). B) Statistical chart of 24‐h fluorescence data for mitochondria internalized by BMDMs in (C) (n=6, mean ± SD). C) Real‐time fluorescence imaging of live cells shows the process of mitochondria uptake by BMDMs (green: Dil, red: Mito Tracker Red, scale bar = 50µm, BMDMs content is 1*10^5^/well, mitochondrial content is 8*10^4^/well). D) Fluorescence labeling diagram of BMDMs in the control group and in the internalized exogenous mitochondria group. E) Confocal images showed the morphological changes of BMDMs in the control group and internalized exogenous mitochondria group (blue: DAPI, red: Mito Tracker Red, green: Mito Tracker green, scale bar = 20µm). F) The mRNA expression of M2 surface markers CD206, CD163, Arg‐1, and anti‐inflammatory indicator IL‐10 in the control group and internalized exogenous mitochondria group were detected by q‐PCR (n=6, mean ± SD, t tests, ^*^ *p*<0.05, ^**^ *p* < 0.01, ^***^ *p* <0.001, and ^****^ *p* <0.0001). control=BMDM, mito=BMDM+mito.

To evaluate the effects of MT on macrophages, we established a transfer system by incubating mitochondria with bone marrow‐derived macrophages (BMDMs). The isolated mitochondria from the hearts of C57BL/6J mice were stained with TMRE (Tetramethylrhodamine Ethyl Ester) to assess mitochondrial membrane potential. Flow cytometry analysis confirmed normal levels, indicating intact mitochondrial function (Figure S1A,B, Supporting Information). Mitochondria were then labeled with MitoTracker Red to facilitate visualization. To determine the optimal concentration of mitochondria, BMDMs (1×10^5^ cells) were incubated with mitochondrial suspensions of varying concentrations (1×10^4^, 2×10^4^, 4×10^4^, 8×10^4^, 16×10^4^, and 32×10^4^). Mitochondrial transfer efficiency was monitored in real‐time for 24 h using a live‐cell imaging system (**Figure** **1**A). The results showed a progressive increase in mitochondrial uptake from 0 to 8 h, reaching a peak and plateauing between 8 and 24 h. Fluorescence intensity analysis revealed that the most efficient mitochondrial uptake occurred at a mitochondrial concentration of 8×10^4^ (Figure 1B,C). While maximal mitochondrial internalization was achieved after 8 h of incubation, equivalent ATP generation was achieved at 2 h. Moreover, the BMDMs exhibited higher mitochondrial membrane potential, and the ROS remained within the physiological compensatory range at the 2‐h time point (Figures S1E,F and S2C,D,F, Supporting Information). Besides, at the optimal 8×10⁴/well concentration, BMDMs had only mildly elevated ROS after 2 h incubation compared with the control, remaining within the physiological compensatory range. However, exceeding this concentration caused ROS to surge beyond cellular compensatory capacity (Figure S2A,B, Supporting Information). Therefore, incubating BMDMs with the optimal mitochondrial concentration (8×10⁴/well) for 2 h represents the most suitable experimental condition.

To delineate the translocation of mitochondria into BMDMs, endogenous mitochondria were labeled with MitoTracker Green, and exogenous mitochondria were labeled with MitoTracker Red (Figure 1D). Following mitochondrial co‐culture, BMDMs exhibited co‐localization of exogenous red‐labeled mitochondria with endogenous green‐labeled mitochondria. The BMDMs that internalized exogenous mitochondria adopted a spindle‐shaped morphology, characteristic of M2‐like macrophages, whereas untreated control BMDMs retained an oval morphology (Figure 1E).

To further investigate the role of MT in BMDM polarization, quantitative PCR (qPCR) analysis was performed to assess the expression of known M2‐associated cytokines. Compared with control BMDMs, cells that internalized exogenous mitochondria showed significantly increased expression of M2 surface markers (arginase‐1 [Arg‐1], cluster of differentiation [CD]206, and CD163) and anti‐inflammatory cytokines (interleukin [IL]‐10) (Figure 1F). Conversely, the expression of pro‐inflammatory cytokines (tumor necrosis factor‐alpha [TNF‐α] and IL‐1β), as well as known M1 surface markers (inducible nitric oxide synthase [iNOS] and CD80), was downregulated in BMDMs following MT compared with the control cells (Figure S3, Supporting Information). To further elucidate the role of MT in promoting M2 polarization of BMDMs, we performed flow cytometry and Western blot analyses to quantify classical M2 macrophage surface markers. Flow cytometry analysis revealed a significant increase in the proportion of M2‐type macrophages (CD206+CD86‐) in the Mito group compared to the Control, reaching levels comparable to the IL‐4 group (Figure S4A–C, Supporting Information). Western blot further demonstrated markedly elevated expression of classic M2 macrophage surface markers (Arg‐1 and CD206) in the Mito group relative to Control, with expression levels essentially equivalent to those observed in the IL‐4 group (Figure S4D,E, Supporting Information). These findings collectively indicate that MT effectively promotes BMDMs polarization toward the M2 phenotype, with an efficacy similar to IL‐4 induction.

### 2.2 MT Enhances the Reparative Functions of Macrophages

**Figure 2:**
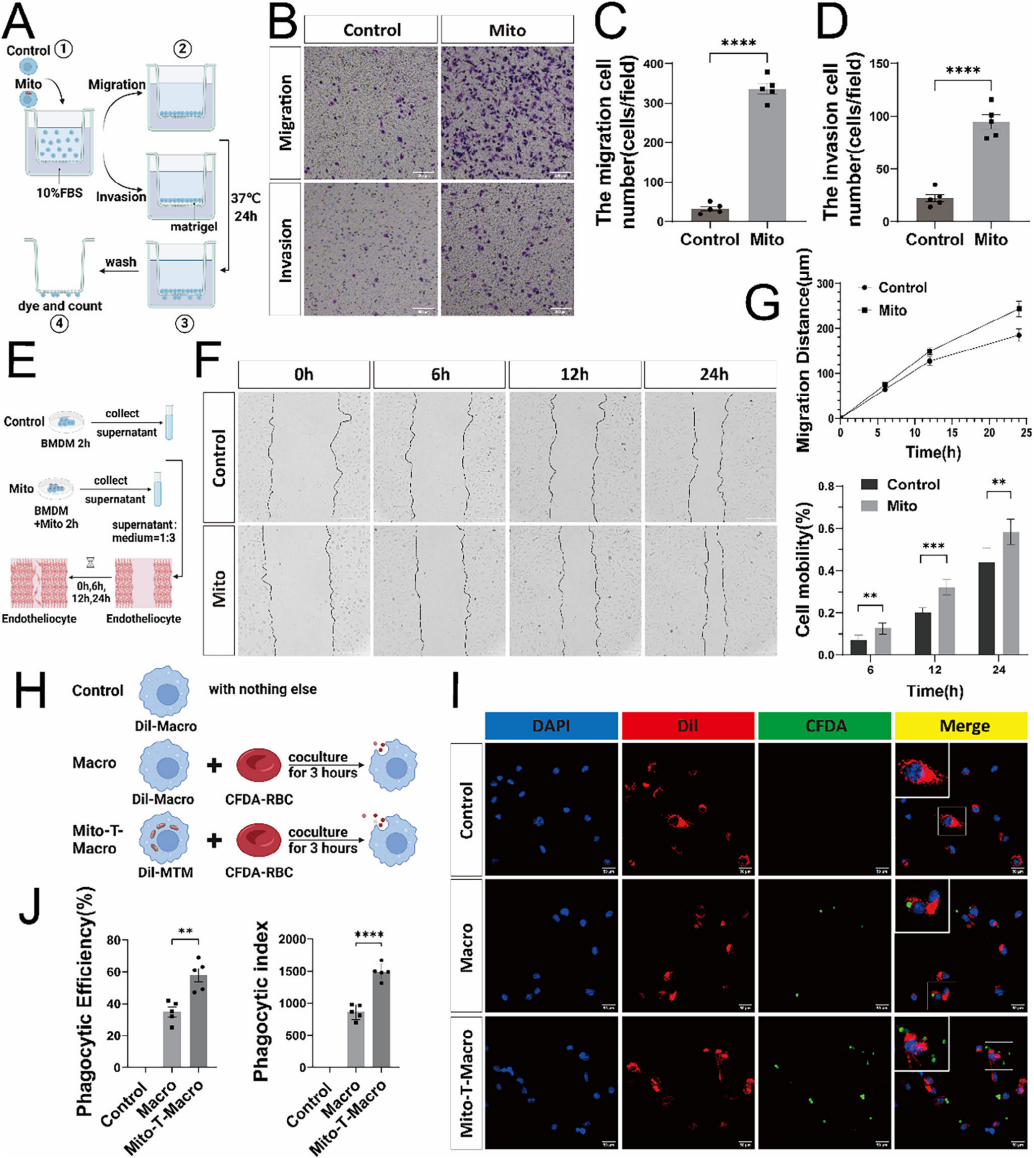
MT enhances the reparative functions of macrophages. A) Experimental schematic of the migration and invasion of BMDMs in the Transwell system for both the control group and the internalized exogenous mitochondria group. B) Microscopic images illustrating the migration and invasion of both control BMDMs and internalized exogenous mitochondria BMDMs after 24 h (scale bar=200µm). C) Statistical graph depicting the number of migrations in (B) (n=5, mean ± SD, one‐way ANOVA, Tukey's multiple comparisons, ^****^ *p* <0.0001). D) Statistical graph depicting the number of invasions in (B) (n=5, mean ± SD, one‐way ANOVA, Tukey's multiple comparisons, ns not significant ^****^ *p* <0.0001). E) The schematic diagram illustrates the procedure for collecting BMDMs cell culture supernatant utilized in the scratch assay for both the control group and the internalized exogenous mitochondria group. F) Scratch assays showing that endotheliocytes exhibited healing at 0‐, 6‐, 12‐, and 24‐h post‐treatment with the cell culture supernatant from both the control group and the internalized exogenous mitochondria group (scale bar=300µm). G) Migration distances and cell mobility of endotheliocytes in the control group and the internalized exogenous mitochondria group at 0, 6, 12, and 24 h in (F) (n=5, mean ± SD, t tests, ^**^ *p* < 0.01, ^***^ *p* <0.001). H) Schematic diagram of the process employed for the phagocytosis experiment in the control group, Macro group, and Mito‐T‐Macro group. I) Confocal microscopy reveals images of the control group, macrophage group, and Mito‐T‐Macro group labeled with Dil following a 3‐h co‐culture with CFDA‐labeled apoptotic erythrocytes (the control group did not receive any apoptotic erythrocytes, blue: DAPI, red: Dil, green: CFDA, scale bar =50µm). J) Phagocytosis efficiency represents the percentage of CFDA+ cells in each group in (I). Phagocytic index represents the CFDA content in positive cells of each group in (I) (n=5, mean ± SD, t tests, ^**^ *p* < 0.01 and ^****^ *p* <0.0001).

To determine the reparative role of BMDMs following MT, the migratory and invasive capacities of BMDMs were evaluated using a Transwell system. The upper chambers were seeded with either control BMDMs or BMDMs pre‐treated with exogenous mitochondria at a density of 1×10^6^ cells mL^−1^ in DMEM supplemented with 10% fetal bovine serum. The cells were then incubated at 37 °C for 24 h. After incubation, non‐migrated cells on the upper side of the membranes were gently removed. The membranes were then fixed and stained, and the migrated cells on the lower membrane surfaces were counted under a microscope (**Figure** **2**A). The average number of migrated BMDMs in the control group was ≈30 cells/field, whereas that of the MT group was ≈330 cells/field, representing a ≈10‐fold increase.

Similarly, the 24‐h invasion assay revealed ≈20 cells/field in the control group compared with ≈100 cells/field in the MT group, representing a ≈5‐fold increase (Figure 2B–D). These findings were further validated in a scratch assay, showing that BMDMs in the MT group exhibited substantially greater migration distances over a 24‐h period compared with those in the control group (Figure 2E–G). Furthermore, the cell mobility rates at 6, 12, and 24 h were markedly higher in the MT group than in the control group (Figure 2G).

To assess the phagocytic function of BMDMs, control and mitochondria‐treated BMDMs were incubated with carboxyfluorescein diacetate (CFDA)‐labeled apoptotic erythrocytes for 3 h (Figure 2H). Phagocytic efficiency was quantified as the percentage of CFDA^+^ cells, whereas the phagocytic index was determined by measuring the CFDA fluorescence intensity within these CFDA^+^ cells. BMDMs in the MT group exhibited significantly higher values in both phagocytic efficiency and index compared with those in the control group, indicating enhanced phagocytic capacity (Figure 2I,J). Collectively, these findings indicated that MT augmented both the migratory and phagocytic capacities of BMDMs in vitro, thereby enhancing their overall reparative function.

### 2.3 MTM Therapy Improves Cardiac Function in Mice Following MI

**Figure 3:**
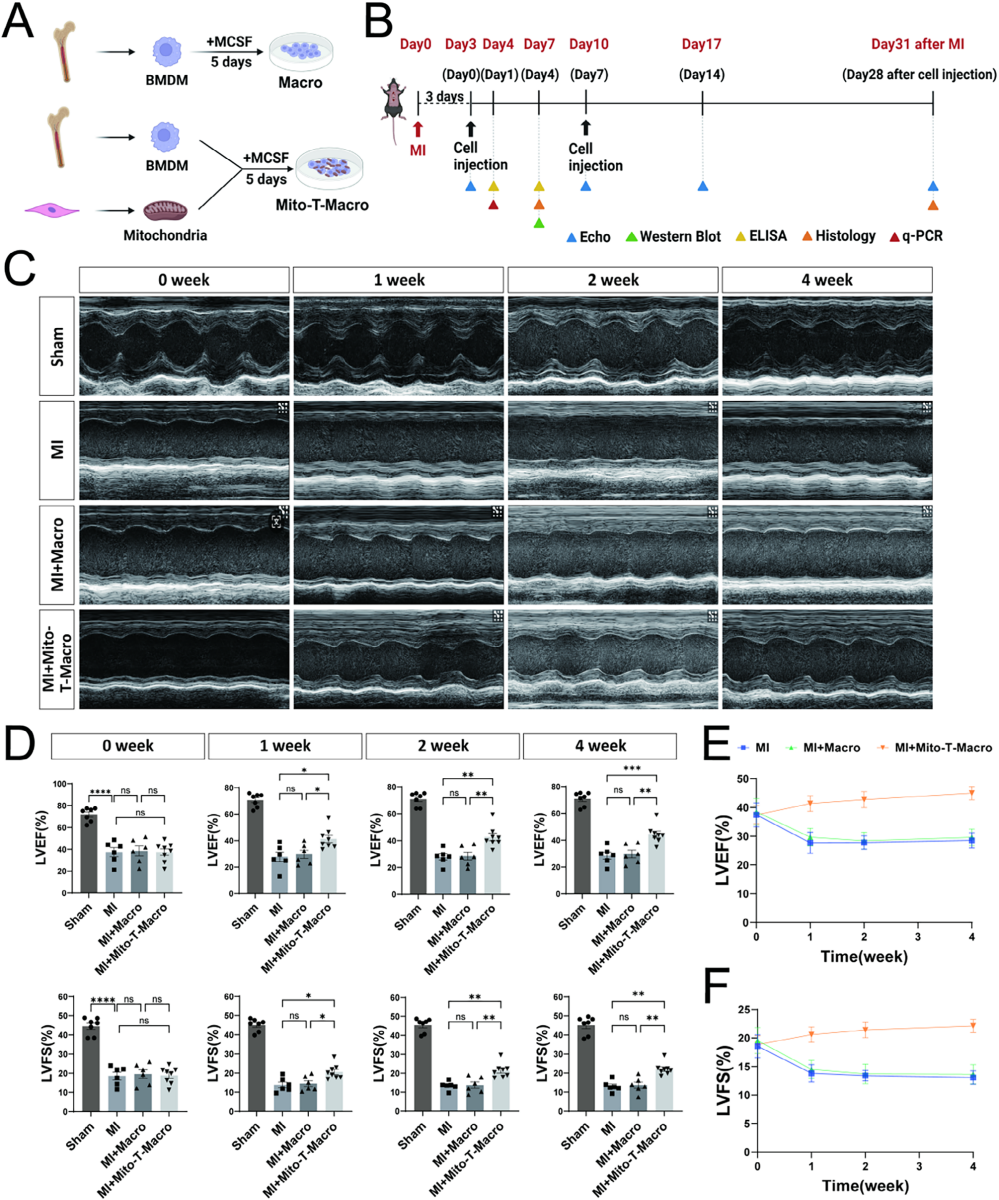
MTM therapy improves cardiac function in mice following MI. A) Schematic diagram of BMDMs for ‘Macro group’ and BMDMs co‐incubated with mitochondria for ‘Mito‐T‐Macro group’. B) Experimental flow chart of the Macro group and Mito‐T‐Macro group for the treatment of MI in mice. C) Representative echocardiographic images (long‐axis view) of mice in Sham, MI, MI+Macro, and MI+Mito‐T‐Macro groups at 0, 1, 2, and 4 weeks after MI. D) Quantitative statistical analysis of left ventricular ejection fraction (LVEF), left ventricular fractional shortening (LVFS) for each group presented in Figure 3C (n=6‐8, mean ± SEM, one‐way ANOVA, Tukey's multiple comparisons, ^*^ *p* < 0.05, ^**^ *p* < 0.01, and ^***^ *p* <0.001). E, F) Line plots illustrating the changes in LVEF and LVFS of the MI, MI+Macro, and MI+Mito‐T‐Macro groups from 0 to 4 weeks post‐MI.

The uptake of exogenous mitochondria by BMDMs enhanced their migratory and phagocytic capacities in vitro, indicating their polarization toward an M2‐like reparative phenotype. We investigated the potential cardioprotective effects of mitochondria‐treated BMDMs in vivo using a murine model of MI. Cell suspensions of control macrophages (Macros) and mitochondria‐treated macrophages (Mito‐T‐Macros) were prepared at a concentration of 2×10^6^ cells mL^−1^ (**Figure** **3**A). Subsequently, mice were intravenously injected with either the Macro or Mito‐T‐Macro cell suspension on post‐MI days 3 and 10 (Figure 3B). Mice within the sham and MI groups were injected with equivalent volumes of saline.

Cardiac function was evaluated at baseline (week 0) and at post‐MI weeks 1, 2, and 4. As expected, the left ventricular ejection fraction (LVEF) and left ventricular fractional shortening (LVFS) values were significantly reduced in the MI group compared with the sham group at all time points. No significant improvements in these parameters were observed in the MI+Macro group at post‐MI weeks 1, 2, and 4 (Figure 3C,D). In contrast, marked functional recovery was observed in the MI+Mito‐T‐Macro group, with LVEF values increasing by ≈19.3% and 23.4% at post‐MI weeks 2 and 4, respectively, compared with the MI group. Additionally, LVFS values increased by ≈18.6% and ≈22.5% at post‐MI weeks 2 and 4, respectively (Figure 3E,F). Additionally, we compared the effects of IL‐4‐pretreated macrophage therapy and MTM therapy on cardiac function in mice at 2 weeks post‐MI. The MI group showed progressive decline in cardiac function, with the LVEF and LVFS values decreased by ≈26.7% and ≈26.2% at 2 weeks compared with baseline. While the MI+IL‐4‐Macro group showed modest improvement (+7.3% LVEF, +8.7% LVFS), and MI+Mito‐T‐Macro group demonstrated significant recovery (+20.7% LVEF, +19.8% LVFS) (Figure S5A–D, Supporting Information). These findings indicated that BMDMs pre‐treated with exogenous mitochondria significantly enhanced cardiac functional recovery following MI.

### 2.4 MTM Therapy Improves Left Ventricular Remodeling in Mice Following MI

**Figure 4:**
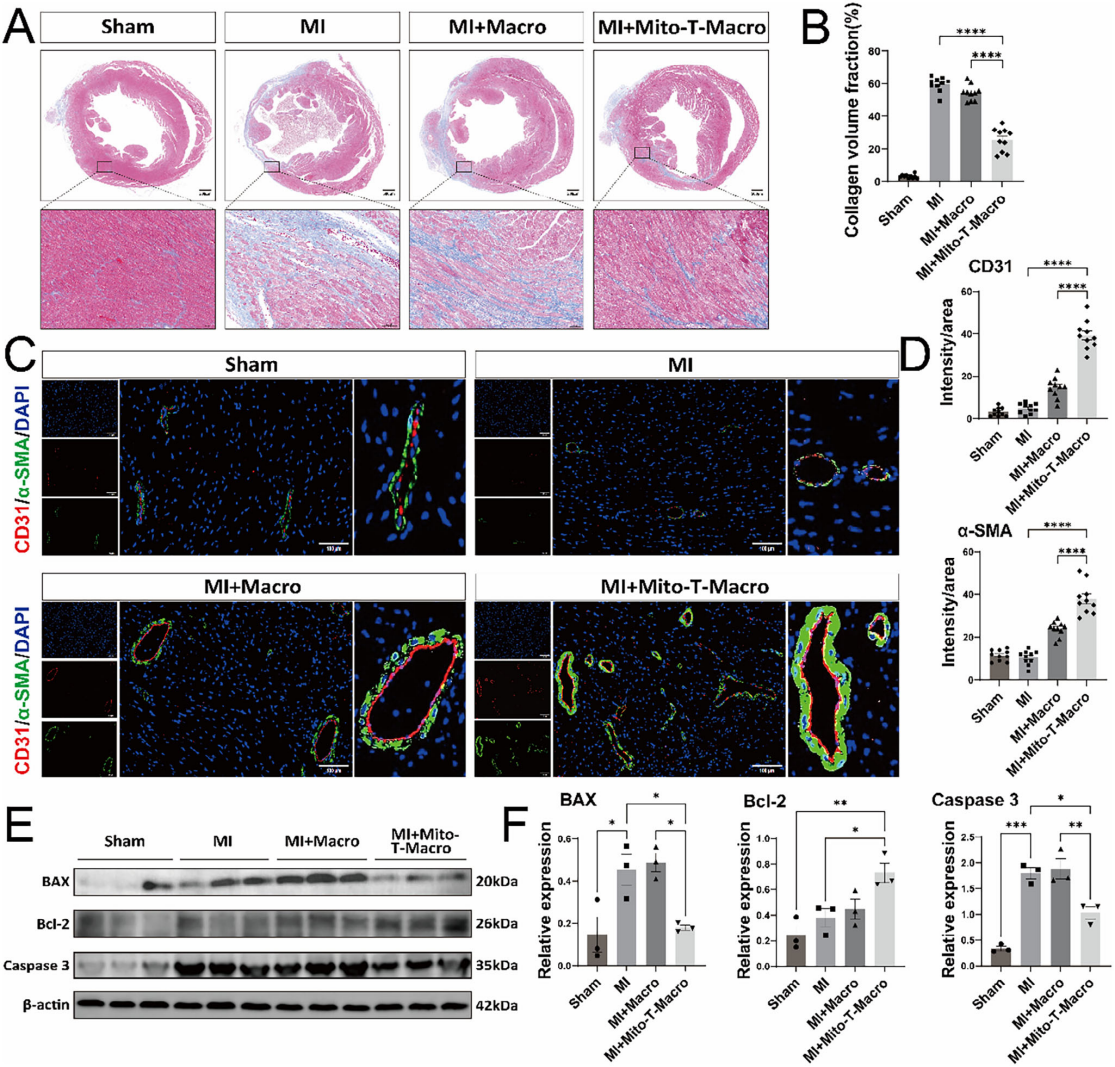
MTM therapy improves left ventricular remodeling in mice following MI. A) Representative images of Masson Trichrome staining of mouse hearts in the Sham, MI, MI+Macro, and MI+Mito‐T‐Macro groups at week 4 after MI. Blue signals indicate the fibrotic areas. Scale bar=500 µm. B) The fibrotic volume proportion in each group of heart tissue specimens in Figure 4A (n=10, mean±SEM, one‐way ANOVA, Tukey's multiple comparisons). C) Representative immunofluorescence images of mouse hearts costained with CD31/PECAM‐1(platelet endothelial cell adhesion molecule‐1; red), α‐SMA (α‐smooth muscle actin; green), and DAPI (blue) in the Sham, MI, MI+Macro, and MI+Mito‐T‐Macro groups at week 4 after MI. Scale bar=100 µm. D) Quantification of CD31+ andα‐SMA+ staining in each group of heart tissue specimens in Figure 4C (n=10, mean±SEM, one‐way ANOVA, Tukey's multiple comparisons). E) Representative immunoblot images of BAX, Bcl‐2, and Caspase‐3 proteins from the injured myocardium in each group of mice on Day 7 post‐MI. β‐actin was used as an internal control. F) Densitometric quantification of BAX, Bcl‐2, and Caspase‐3 proteins in each group of Figure 4E (n=3, mean±SEM, one‐way ANOVA, Tukey's multiple comparisons). ^*^ *p* < 0.05, ^**^ *p* < 0.01, ^***^ *p* <0.001, and ^****^ *p* <0.0001.

To determine the effects of mitochondria‐treated BMDMs on cardiac remodeling in vivo, we analyzed myocardial fibrosis, angiogenesis, and apoptosis in mice following MI. Hematoxylin and eosin (H&E) and Masson's trichrome staining were used to analyze cardiac fibrosis at post‐MI week 4 (**Figure** **4**A). Compared with the sham group, the MI group exhibited a notable increase in scar tissue and fibrotic area. The extent of fibrosis in the MI+Macro group was similar to that observed in the MI group. However, the MI+Mito‐T‐Macro group exhibited a markedly reduced fibrotic area relative to both the MI+Macro and MI groups (Figure 4B). Simultaneously, we also compared the effects of IL‐4‐pretreated macrophage therapy and MTM therapy on cardiac fibrosis in mice at 2 weeks post‐MI. Compared with the Sham group, the MI group exhibited a notable increase in scar tissue and fibrotic area. However, the MI+Mito‐T‐Macro group exhibited a markedly reduced fibrotic area relative to both the MI+IL‐4‐Macro and MI groups (Figure S5E,F, Supporting Information).

Furthermore, immunofluorescence staining of cardiac tissue was performed using the angiogenesis marker CD31 and the arteriogenesis marker alpha‐smooth muscle actin (α‐SMA) to evaluate the effects of mitochondria‐treated BMDMs on cardiac vascular regeneration. Compared with the MI and MI+Macro groups, the MI+Mito‐T‐Macro group exhibited significantly increased expression of both CD31 and α‐SMA, indicating enhanced angiogenesis and arteriogenesis (Figure 4C,D).

Additionally, apoptosis was assessed in the injured myocardium of the mice on post‐MI Day 7. Compared with the MI group, the MI+Mito‐T‐Macro group showed markedly reduced apoptosis, as evidenced by significant downregulation of the pro‐apoptotic markers, BCL‐2‐associated X protein (BAX) and caspase‐3, along with a notable upregulation of the anti‐apoptotic protein, B‐cell lymphoma‐2 (BCL‐2) (Figure 4E,F). These findings indicated that MTM therapy substantially improved ventricular remodeling after MI by promoting angiogenesis and inhibiting fibrosis and cardiomyocyte apoptosis.

### 2.5 MTM Therapy Facilitates the Early Differentiation and Continuous Infiltration of CD206^+^ Reparative Macrophages in Mice Following MI

**Figure 5:**
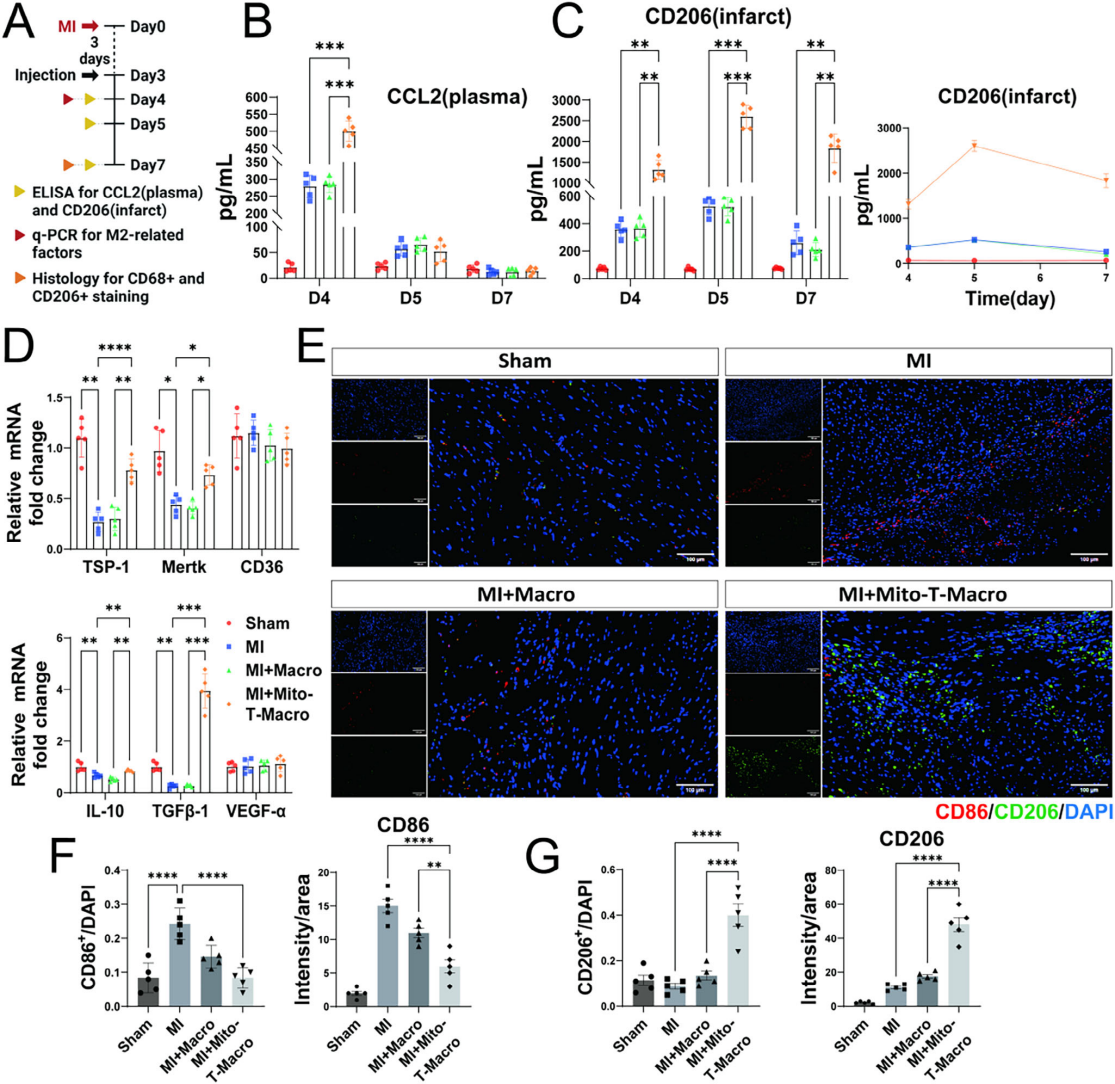
MTM therapy facilitates the early differentiation and continuous infiltration of CD206+ reparative macrophages in mice following MI. A) Timeline of sample collection for Sham, MI, MI+Macro, and MI+Mito‐T‐Macro groups after MI. B) The concentration of CCL2 in serum was measured using ELISA on Days 4, 5, and 7 for each group post‐MI (n=5, mean±SEM, one‐way ANOVA, Tukey's multiple comparisons, unit: pg mL^−1^). C) The concentration of CD206 at the infarct area of myocardial tissue was measured by ELISA on Days 4, 5, and 7 for each group post‐MI (n=5, mean±SEM, one‐way ANOVA, Tukey's multiple comparisons, unit: pg mL^−1^). D) mRNA levels of genes involved in anti‐inflammatory responses (IL‐10, TGF‐β1, and VEGF‐α) and cell adhesion (TSP‐1, Mertk, and CD36) at the infarct area of myocardial tissue on Day 4 post‐MI (n=5, mean±SEM, one‐way ANOVA, Tukey's multiple comparisons). E) Representative immunofluorescence images of the myocardial injury area in each group on Day 7 post‐MI demonstrated the infiltration and distribution of CD86‐positive and CD206‐positive cells (blue: DAPI, red: CD86, green: CD206; Scale bar = 100 µm). F) Quantitative analysis of percentage of CD86‐positive cells per nuclei (left) and CD86‐positive staining (mean fluorescence intensity of CD86‐positive cells) in the myocardial injury area (right) in Figure 5E. *n* = 5, mean±SEM, one‐way ANOVA, Tukey's multiple comparisons. G) Quantitative analysis of percentage of CD206‐positive cells per nuclei (left) and CD206‐positive staining (mean fluorescence intensity of CD206‐positive cells) in the myocardial injury area (right) in Figure 5E. *n* = 5, mean±SEM, one‐way ANOVA, Tukey's multiple comparisons. ^*^ *p* < 0.05, ^**^ *p* < 0.01, ^***^ *p* <0.001, and ^****^ *p* <0.0001.

To elucidate the regulatory mechanisms by which MTMs impact cardiac remodeling, serum levels of the monocyte chemoattractant C‐C motif chemokine ligand 2 (CCL2) and the expression of CD206 in the infarct areas of mice were assessed at post‐MI days 4, 5, and 7 (**Figure** **5**A). Compared with the sham group, CCL2 expression was significantly elevated in the MI group at post‐MI day 4, indicating robust recruitment of circulating monocytes to the injured myocardium. Furthermore, serum CCL2 levels in the MI+Mito‐T‐Macro group were 1.5‐fold higher than those in the MI and MI+Macro groups, suggesting that MTMs enhanced macrophage mobilization to augment the local immune response in the infarcted heart (Figure 5B). In response to elevated CCL2, CD206^+^ reparative macrophages were detected in the injured myocardium of all mice, emerging on post‐MI day 4, peaking on post‐MI day 5, and diminishing by post‐MI day 7. However, the MI+Mito‐T‐Macro group exhibited a markedly higher abundance of CD206^+^ macrophages, ≈3‐fold higher than that of the MI and MI+Macro groups on day 4. This difference increased to ≈5‐fold on day 5 and approached ≈10‐fold by day 7 (Figure 5C). These findings indicated that MTMs significantly promoted the early differentiation of macrophages into the M2‐like reparative phenotype, predominantly characterized by CD206 expression. Furthermore, the elevated presence of CD206^+^ macrophages in the infarct areas of the mice within the MI+Mito‐T‐Macro group was sustained at a high level from post‐MI day 4 to post‐MI day 7.

To further validate the substantial increase in CD206 expression induced by MTMs on post‐MI day 4, we examined the mRNA expression of key factors associated with M2 macrophage polarization in the injured myocardium. These factors included cell adhesion molecules (thrombospondin‐1 [TSP‐1], Mer tyrosine kinase [MERTK], and CD36) as well as anti‐inflammatory mediators (IL‐10, transforming growth factor‐beta 1 [TGF‐β1], and vascular endothelial growth factor‐alpha [VEGF‐α]). Compared with the MI group, the MI+Mito‐T‐Macro group exhibited significantly elevated expression of *Tsp‐1* (3‐fold)*, Mertk* (2‐fold)*, Il‐10* (1.5‐fold), and *Tgf‐β1* (20‐fold) (Figure 5D).

The abundance of CD206^+^ macrophages in the MI group was markedly reduced on post‐MI day 7, whereas it was significantly higher in the MI+Mito‐T‐Macro group. Thus, the effects of MT on BMDM polarization were further evaluated at this time point. CD86 and CD206 staining were performed to distinguish between pro‐inflammatory and reparative macrophages, respectively. A significant increase in the number of CD86^+^ macrophages was detected in the myocardium of mice within the MI group compared with the sham group. However, a notable reduction in the number of CD86^+^ macrophages, as well as the extent of their infiltration, was observed in the MI+Mito‐T‐Macro group compared with the MI group (Figure 5E,F). In contrast, a significant increase in the number of reparative CD206^+^ macrophages was observed in the Mito‐T‐Macro group, with an infiltration area larger than that of the MI group (Figure 5E,G).

These differences in CD86 and CD206 detection indicated that MTMs promoted the infiltration and sustained presence of reparative CD206^+^ macrophages while attenuating the accumulation of pro‐inflammatory CD86^+^ macrophages on post‐MI day 7. Collectively, these findings demonstrated that MTMs enhanced the recruitment of monocytes to the injured myocardium and facilitated their early differentiation into reparative CD206^+^ macrophages while maintaining a prolonged reparative macrophage presence at the injury site.

### 2.6 Exogenous Mitochondria Internalized by BMDMs are Transferred to Cardiomyocytes

**Figure 6:**
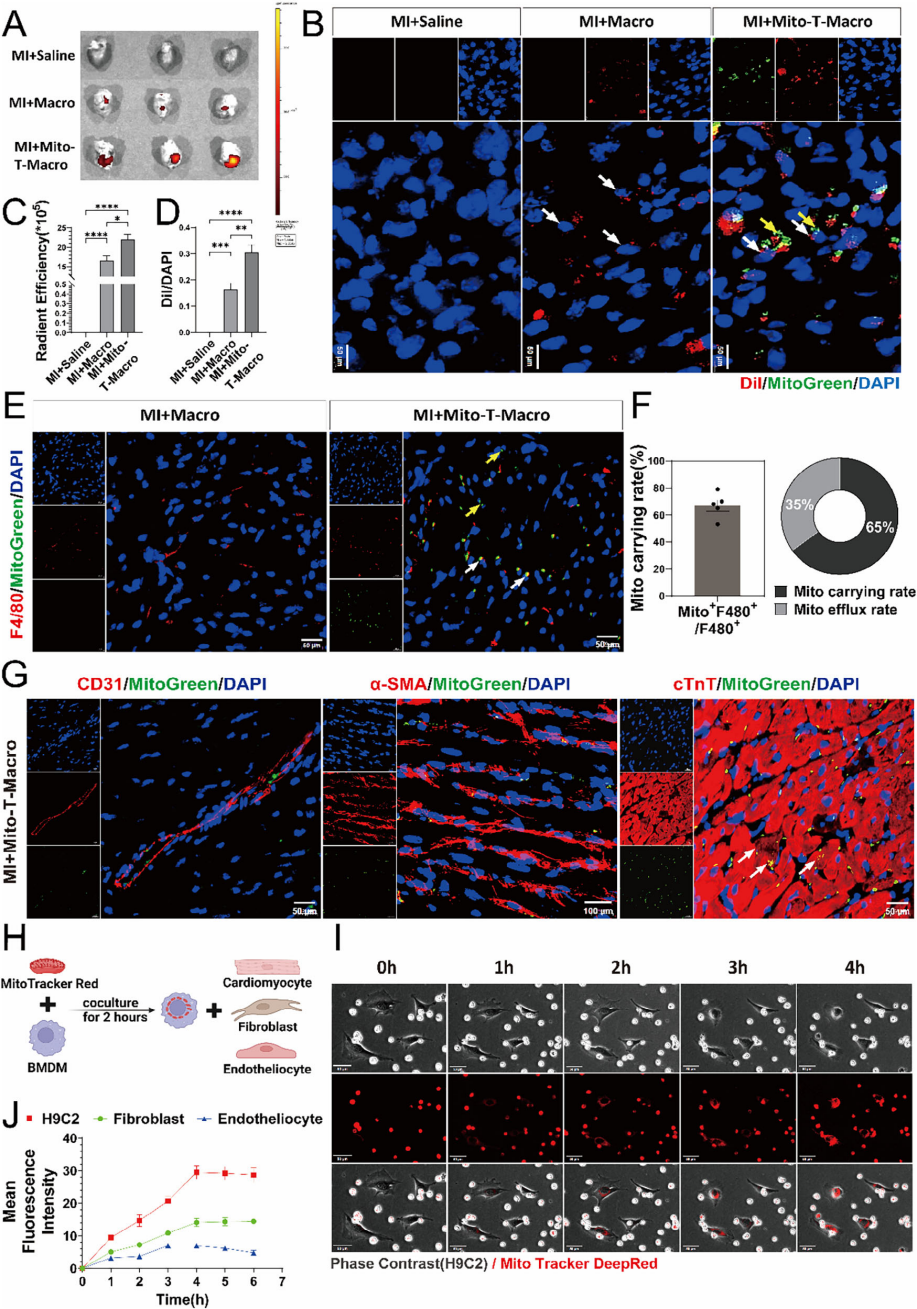
Exogenous mitochondria internalized by BMDMs are transferred to cardiomyocytes. A) On the third day following MI, mice were administered saline (MI+Saline), Dil‐labeled macrophages (MI+Macro), and Dil‐labeled Mito‐T‐Macro (MI+Mito‐T‐Macro) via tail vein injection. Isolated heart imaging was conducted 8–12 h post‐injection for all groups. B) The representative immunofluorescence images of the hearts from each group depicted in Figure 6A above illustrate Dil‐labeled macrophages infiltrating the infarct area of myocardial tissue (Negative control: The MI+Saline group; blue: DAPI, red: Dil, green: MitoTracker Green; yellow arrows indicate exogenous mitochondria, white arrows indicate Dil‐labeled macrophages; Scale bar = 50µm). C) Fluorescence quantitative analysis of ex vivo heart imaging for each group in Figure 6A (n=5, mean±SEM, one‐way ANOVA, Tukey's multiple comparisons). D) Quantitative analysis of cardiac immunofluorescence images for each group in Figure 6B (n=5, mean±SEM, one‐way ANOVA, Tukey's multiple comparisons). E) Representative immunofluorescence images of the MI+Macro group and the MI+Mito‐T‐Macro group illustrate the distribution of macrophages within the injured myocardium on Day 3 post‐MI. Notably, exogenous mitochondrial signals were observed in the MI+Mito‐T‐Macro group (blue: DAPI, red: F4/80, green: Mito Tracker Green; yellow arrows indicate free mitochondria out of macrophages, white arrows indicate exogenous mitochondria co‐located with macrophages; Scale bar = 50µm). F) Quantitative analysis was conducted on the mitochondria carrying rate of macrophages(left) as well as the distribution ratio of mitochondria both intra‐ and extracellularly within macrophages(right), as depicted in Figure 6E. G) Representative immunofluorescence images illustrate the distribution of mitochondria in CD31+ endotheliocytes, α‐SMA+ fibroblasts, and cTnT+ cardiomyocytes within the MI+Mito‐T‐Macro group (blue: DAPI, red: CD31/α‐SMA/cTnT, green: Mito Tracker Green; white arrows indicate exogenous mitochondria were untaken by cardiomyocytes; Scale bar =50µm/100µm /50µm). H): The flow chart illustrated that in vitro BMDMs were co‐cultured with endotheliocytes, fibroblasts, and cardiomyocytes to investigate mitochondrial uptake. I) Real‐time fluorescence imaging of representative living cells captured time‐lapse images of mitochondrial‐labeled BMDMs co‐cultured with cardiomyocytes at 0, 1, 2, 3, and 4 h. J) Quantitative analysis was conducted on the mean fluorescence intensity of mitochondria uptake in endotheliocytes, fibroblasts, and cardiomyocytes at various time points. ^*^ *p* < 0.05, ^**^ *p* < 0.01, ^***^ *p* <0.001, and ^****^ *p* <0.0001.

To evaluate the targeting efficiency of MTMs to the injured myocardium, exogenous mitochondria were labeled with MitoTracker Green, and BMDMs were labeled with 1,1'‐dioctadecyl‐3,3,3',3'‐tetramethylindocarbocyanine perchlorate (Dil). Mice were administered either saline (Saline group), Dil‐labeled BMDMs (Macro group), or Dil‐labeled mitochondria‐treated BMDMs (Mito‐T‐Macro group) via tail vein injection on post‐MI day 3. After 12 h, small‐animal in vivo fluorescence imaging was performed to visualize the labeled BMDMs in the mouse hearts, and the fluorescence intensities were quantified. The results showed no detectable Dil fluorescence signal in the control saline‐injected mice and a weak signal in the mice within the Macro group. However, a markedly enhanced fluorescence signal was detected in the mice within the Mito‐T‐Macro group (**Figure** **6**A,C). We subsequently assessed the immune activation following systemic administration of BMDMs and their biodistribution throughout the body. ELISA results demonstrated elevated serum TNF‐α levels in both MI and MI+Mito‐T‐Macro groups compared to the Sham. Importantly, the MI+Mito‐T‐Macro group showed significantly reduced TNF‐α concentrations relative to the MI group alone, suggesting that MTM therapy not only avoided eliciting immune rejection but also ameliorated systemic inflammation. Similar patterns were observed for IFN‐γ and IL‐1β (Figure S6A, Supporting Information). Besides, in vivo imaging results demonstrated that MTM exhibited the highest distribution in the heart, followed by the liver, kidneys, and lungs, with no detectable accumulation in the spleen. Compared to the MI+Macro group, MTM therapy showed enhanced targeting efficiency to the heart while significantly reducing biodistribution leakage in pulmonary and renal tissues (Figure S6B,C, Supporting Information).

Subsequently, co‐localization of DiI and MitoTracker Green fluorescence was used to visualize the spatial distribution of mitochondria‐treated, Dil‐labeled BMDMs in myocardial tissue sections. Indeed, a significant increase in Dil‐positive BMDMs was observed in the MI+Mito‐T‐Macro group. Moreover, MitoTracker Green‐labeled mitochondria were found closely associated with the inner surface of the macrophage membrane, indicating successful translocation of the mitochondria‐treated macrophages into the injured myocardium following MI (Figure 6B,D).

To track the fate of transplanted mitochondria within macrophages, immunofluorescence staining was performed using F4/80 to label macrophages and MitoTracker Green to label mitochondria. Interestingly, MitoTracker Green signal detection showed ≈65% co‐localization with F4/80^+^ cells, indicating that around 35% of the transplanted mitochondria were no longer retained within macrophages (Figure 6E,F). This observation raised the question of where these released mitochondria had translocated. To investigate this, we examined the three predominant cell types in the myocardium: CD31⁺ endotheliocytes, α‐SMA⁺ fibroblasts, and cTnT⁺ cardiomyocytes. The results revealed that the exogenous mitochondria primarily localize within cardiomyocytes, suggesting potential mitochondrial transfer‐mediated crosstalk between macrophages and cardiomyocytes (Figure 6G).

To further validate these observations, in vitro experiments were conducted. BMDMs were incubated with MitoTracker Red‐labeled mitochondria for 2 h and subsequently co‐cultured with endotheliocytes, fibroblasts, or H9C2 (Figure 6H). The co‐culture systems were continuously monitored using live‐cell real‐time imaging to assess mitochondrial transfer dynamics. Fluorescence signals progressively increased in all recipient cell types, beginning at 1 h, reaching a plateau by 4 h, and remaining stable thereafter (Figure 6I; Figure S7, Supporting Information). Among the three cell types, H9C2 cells exhibited the highest fluorescence intensity, followed by fibroblasts, whereas endotheliocytes displayed the weakest signal (Figure 6J). Altogether, these findings demonstrated that the MTMs selectively localized to the injured myocardium and spontaneously transferred their internalized mitochondria to cardiomyocytes.

### 2.7 Transcriptomic Profiling Reveals Mitochondrial Transfer Improves Post‐MI Cardiac Recovery via Metabolic Reprogramming and Oxidative Stress Mitigation

**Figure 7:**
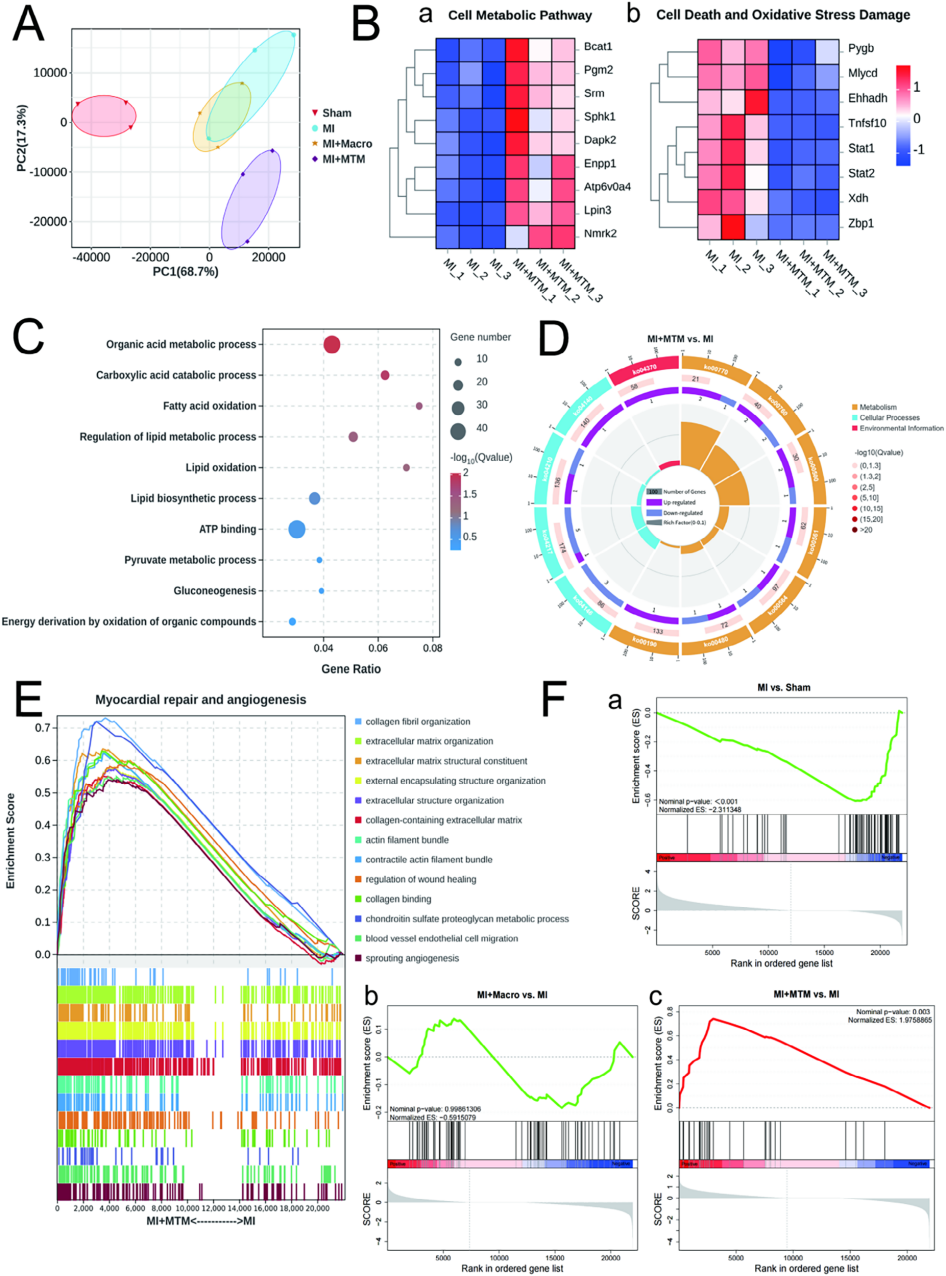
Transcriptomic profiling reveals mitochondrial transfer improves post‐MI cardiac recovery via metabolic reprogramming and oxidative stress mitigation. A) Principal component analysis (PCA) by transcriptional analysis reveals in‐group clusters with minimal overlap. B) The clustering heat map shows the differentially expressed genes related to cell metabolism, cell death, and oxidative stress pathways between the MI+MTM group and the MI group (|log2FC| > 2, FDR < 0.05). C) Top 10 enriched gene ontology biological process (GO‐BP) terms of genes significantly downregulated in the MI group and significantly upregulated in the MI+MTM group. D) The KEGG enrichment bubble plot demonstrates other upregulated pathways in the MI+MTM group compared with the MI group, including metabolism, cellular processes, and environmental information. E) Gene set enrichment analysis (GSEA) plots showing the enrichment of gene sets related to myocardial repair and angiogenesis between MI+MTM and MI groups. F) Gene set enrichment analysis (GSEA) plots showing the enrichment of gene sets related to fatty acid β‐oxidation capacity between MI and Sham groups, MI+Macro and MI groups, MI+MTM and MI groups.

To obtain evidence supporting the potential of transferred mitochondria in improving post‐MI cardiac function, we performed RNA sequencing analysis on cardiac tissues from four experimental groups at 4 weeks post‐MI: Sham, MI, MI+Macro, and MI+MTM groups. Principal component analysis (PCA) revealed that samples primarily cluster by the type of treatment. Overlap was observed between the MI and MI+Macro groups, while the MI+MTM group clustered discretely from the MI group (**Figure** **7**A). First, we screened for the most significantly differentially expressed genes between the MI and MI+MTM groups (|log2FC| > 2, FDR < 0.05) based on specific pathways, including cellular metabolism, cell death, and oxidative stress pathways, and generated a clustering heatmap using Z‐score normalized expression values. In the MI+MTM group, Cluster a contained 9 upregulated genes enriched in cellular catabolic pathways (e.g., Enpp1, Atp6v0a4), while Cluster b was associated with downregulation of genes related to cell death and oxidative stress damage (e.g., Stat1/2, Zbp1). This pattern supports the beneficial effects of MTM treatment in promoting myocardial energy recovery and suppressing oxidative stress damage after MI (Figure 7B). Subsequently, gene ontology enrichment analysis identified the most crucial biological processes rescued by MTM. Among the top 10 significantly upregulated pathways by MTM, 6 were associated with fatty acid metabolism, and 4 were related to oxidative phosphorylation processes. These findings indicate that MTM attenuates the adverse metabolic reprogramming induced by MI, primarily through enhancing fatty acid metabolism and oxidative phosphorylation (Figure 7C). Besides, the KEGG enrichment bubble plot demonstrates more comprehensive metabolic improvement pathways mediated by MTM (Figure 7D; Table S1, Supporting Information). We further analyzed gene expression related to cardiac repair and angiogenesis. Gene set enrichment analysis (GSEA) revealed that MTM enhances myocardial extracellular matrix remodeling capacity while significantly improving cardiac repair and angiogenesis (Figure 7E). GSEA analysis further revealed that fatty acid β‐oxidation capacity was downregulated post‐MI, which could not be rescued by Macro treatment but was effectively restored by MTM (Figure 7F). This suggests that the transplanted mitochondria may directly enhance myocardial fatty acid metabolic capacity. In conclusion, RNA sequencing analysis revealed that the transferred mitochondria in cardiomyocytes were significantly correlated with improved myocardial energy metabolism, inhibition of cell death and oxidative stress, and enhanced cardiac repair and angiogenesis. Special attention should be paid to the relationship between mitochondrial transfer and myocardial fatty acid metabolic capacity.

## 3 Discussion and Conclusion

In this study, we demonstrate that MT endows macrophages with a robust reparative capacity in the context of myocardial injury following MI. In vitro, MT naturally induces the polarization of BMDMs toward an M2‐like phenotype, characterized by enhanced reparative functions, including increased migration, invasion, and phagocytosis. In vivo, MTM therapy significantly improves cardiac function and attenuates left ventricular remodeling by reducing fibrosis, suppressing apoptosis, and promoting angiogenesis. Mechanistically, MTM treatment facilitates an accelerated transition of macrophages within the injured myocardium to a reparative phenotype, thereby prolonging the reparative phase and amplifying pro‐healing signaling.

We discovered that MTMs were efficiently targeted to the injured myocardium. Notably, ≈35% of the transplanted mitochondria translocated from the macrophages to cardiomyocytes. These findings highlight the therapeutic potential of mitochondria‐based strategies in promoting macrophage‐mediated tissue repair and improving cardiac function following MI.

The classic M1/M2 polarization paradigm remains a widely used framework for characterizing macrophage phenotypes. M1‐type macrophages, activated by stimuli such as IFN‐γ and LPS, exhibit high surface expression of CD86, CD80, and MHC‐II along with intracellular iNOS production. These cells predominantly secrete pro‐inflammatory cytokines, including IL‐1β and TNF‐α, executing critical roles in host defense through antibacterial and anti‐tumor activities while promoting inflammatory responses. In contrast, M2‐type macrophages polarized by IL‐4 and IL‐13 demonstrate characteristic expression of CD206, CD163, and arginase‐1 (Arg‐1), while producing anti‐inflammatory mediators like IL‐10 and TGF‐β that are essential for tissue remodeling, immunoregulation, and angiogenesis.^[^ ^4^, ^17^ ^]^ Besides, M1 macrophages primarily rely on glycolysis to meet their energetic demands, enabling rapid pro‐inflammatory responses. These responses are characterized by heightened phagocytic activity, robust antigen presentation via major histocompatibility complex (MHC) class‐II molecules, and elevated ROS production. In contrast, M2 macrophages depend on mitochondrial OXPHOS for sustained energy production and are associated with an anti‐inflammatory gene expression profile that promotes tissue repair.^[^ ^9^, ^18^, ^19^, ^20^ ^]^

In vitro, we observed that BMDMs subjected to MT exhibited a characteristic M2‐like spindle‐shaped morphology, whereas cells in the control group retained their original round or oval appearance. Compared with the control group, the Mito‐T‐Macro group showed a significant upregulation of M2‐associated markers, including known surface markers, such as CD206, CD163, ARG‐1, and the cytokine IL‐10. Moreover, flow cytometry and Western blot confirmed that the Mito group showed significantly increased M2 macrophages (CD206+CD86‐) and markers (Arg‐1/CD206), matching IL‐4 group levels. Additionally, MTM demonstrated enhanced reparative functions, such as increased migration, invasion, and phagocytosis.

These findings suggest that MT intrinsically promotes the polarization of BMDMs toward an M2‐like phenotype, independently of exogenous growth factor stimulation (e.g., IL‐4 or IL‐13). Moreover, our study provides compelling evidence that MTM offers distinct therapeutic advantages over conventional IL‐4‐pretreated macrophages for myocardial repair. MTM therapy demonstrated superior functional outcomes, achieving 20.7% and 19.8% improvements in LVEF and LVFS, respectively, at 2 weeks post‐MI, compared to modest 7‐8% gains with IL‐4‐pretreated macrophage therapy. Besides, MTM treatment significantly reduced fibrotic burden, suggesting it not only enhances functional recovery but also actively mitigates adverse remodeling. These results imply that MT confers macrophages with greater phenotypic stability and multifunctional repair capacity, potentially through both immunomodulation and metabolic reprogramming. Future studies should elucidate how MT stabilizes reparative phenotypes of macrophages, positioning MTM as a next‐generation macrophage therapy surpassing cytokine‐based limitations.^[^ ^9^, ^21^ ^]^

Following MI, local immune cells undergo complex and dynamic changes, with particular emphasis on the phenotypic transition of macrophages.^[^ ^2^, ^22^, ^23^ ^]^ Within the first 24 h, post‐MI, infarct‐associated cell death leads to the release of damage‐associated molecular patterns (DAMPs), which recruit and activate circulating monocytes.^[^ ^24^, ^25^ ^]^ These monocytes infiltrate the injured myocardium and differentiate into macrophages, initiating a robust inflammatory response characterized by a surge of pro‐inflammatory cytokines.^[^ ^26^ ^]^ This inflammatory phase typically peaks around post‐MI day 3 and gradually subsides thereafter.^[^ ^27^ ^]^ Concurrently, macrophages begin to adopt a reparative phenotype, promoting collagen deposition, extracellular matrix remodeling, and angiogenesis, all of which generally persist until around post‐MI day 14.^[^ ^17^, ^28^ ^]^ A major finding of this study is that MT promotes the early transition of macrophages toward a reparative phenotype and sustains elevated expression of long‐term tissue repair factors, both of which are critical for myocardial recovery following MI.

Mice receiving MTM treatment exhibited significantly higher expression of the chemokine CCL2 on post‐MI day 4 compared with the control group. This upregulation of CCL2 facilitated the early recruitment of circulating monocytes, potentially accelerating the tissue repair process. Increasing the influx of immune cells capable of efferocytosis and clearing necrotic debris is a crucial step in mitigating inflammation and initiating tissue repair.^[^ ^29^ ^]^ Efferocytosis is a key function of reparative macrophages. Through the degradation of phagocytosed apoptotic cells, this process prevents the presentation of self‐antigens, thereby limiting the production of pro‐inflammatory cytokines and promoting the secretion of pro‐repair mediators.^[^ ^30^ ^]^

We observed a significant increase in CD206 expression within the injured myocardium of mice treated with MTMs between post‐MI days 4 and 7. Notably, by post‐MI day 7, CD206 expression in the Mito‐T‐Macro group was ≈10‐fold higher than that in the control group. Furthermore, the expression of cell adhesion molecules (TSP‐1 and MERTK) and anti‐inflammatory mediators (IL‐10 and TGF‐β1) was significantly upregulated in the Mito‐T‐Macro group as early as post‐MI day 4, suggesting an accelerated emergence of CD206^+^ macrophages in the injured myocardium. Cardiac immunofluorescence analysis revealed sustained infiltration of CD206^+^ macrophages in the infarcted myocardium at post‐MI day 7, indicating delayed regression of the reparative phase. These macrophages exhibit an enhanced efferocytotic capacity, facilitated by the upregulation of TSP‐1 and MERTK, which enable accurate recognition and clearance of apoptotic cells.

Our evidence suggested that MT enhances the migration, invasion, and phagocytic capacities of macrophages (Figure 2). And within 24 h post‐MI, the myocardium releases chemokines (e.g., CCL2) to recruit circulating macrophages, which in turn secrete additional CCL2, establishing a positive feedback loop. MTM amplifies this process by increasing CD206+ macrophage accumulation in the infarct zone, accelerating their recruitment and prolonging tissue retention (Figure 5B,C). Although enhanced DiI fluorescence in the heart of the MI+Mito‐T‐Macro group might suggest increased recruitment of BMDMs, these observations can't substantiate a strict causal relationship between mitochondrial intervention and BMDMs targeting infarcted cardiac tissues yet. Further experimental evidence is required to substantiate the issue of targeting.

Notably, mitochondrial tracking studies demonstrated that ≈65% of exogenously delivered mitochondria co‐localized with F4/80^+^ macrophages in the infarct zone, whereas the remaining ≈35% were subsequently released from the MTMs and internalized by cardiomyocytes. In vitro experiments further confirmed that, within the same co‐culture system, cardiomyocytes preferentially internalized these mitochondria.

This preferential uptake may reflect the high energy demands of the myocardium, particularly under ischemic and hypoxic conditions, where mitochondrial damage is most pronounced in cardiomyocytes.^[^ ^6^, ^31^, ^32^ ^]^ A substantial supply of fully functional mitochondria is essential for maintaining optimal OXPHOS levels, thereby ensuring efficient myocardial contraction and relaxation.^[^ ^33^, ^34^ ^]^ Our previous research has demonstrated that MT enhances cardiac function by augmenting respiratory capacity and energy production.^[^ ^16^, ^35^ ^]^ Therefore, while maintaining their reparative functions, macrophages may transfer residual mitochondria to cardiomyocytes, supporting the latter's energy metabolism and function.

While our data suggest cardiomyocytes internalization of transferred mitochondria, the current study has two key limitations that warrant discussion. First, the absence of direct in vivo visualization leaves the mitochondrial uptake mechanism incompletely resolved—whether this occurs via active endocytosis, membrane fusion, or tunneling nanotubes (TNT) remains speculative. Second, while fluorescence colocalization implies mitochondrial transfer, it cannot exclude potential artifacts from extracellular mitochondrial adherence or passive diffusion. To address these gaps, it is essential to employ transgenic mice with fluorescent protein‐labeled mitochondria for validation.

Besides, aligned with endogenous immune mechanisms, MTMs rapidly respond to DAMPs released at the injury site, facilitating the timely transition of macrophages toward a reparative phenotype in the infarcted myocardium. This contributes to the mitigation of post‐MI damage and promotes tissue repair. Importantly, MTM therapy also addresses two critical limitations associated with conventional MT: (i) the poor viability and function of isolated mitochondria in the high‐calcium extracellular environment^[^ ^36^ ^]^, and (ii) the potential immunogenicity of free mitochondria as organelles exposed to extracellular fluid.^[^ ^37^ ^]^ However, the sourcing of mitochondria remains a major challenge in the development of MTM therapy.

In this study, mitochondria were isolated from adult mouse cardiac tissues. However, to establish a more versatile and scalable MTM platform, it is crucial to explore alternative mitochondrial sources. Chang et al.^[^ ^38^ ^]^ have reported that high‐quality mitochondria can be derived from induced pluripotent stem cells (iPSCs), demonstrating therapeutic potential in models of neurodegenerative diseases, including Parkinson's disease. Similarly, Baldwin et al.^[^ ^39^ ^]^ have shown that mesenchymal stem cells (MSCs) can transfer mitochondria to CD8^+^ T cells via tunneling nanotubes (TNTs), thereby enhancing their anti‐tumor immune activity. These findings highlight iPSCs and MSCs as promising alternative sources for mitochondrial isolation.

Traditional CAR‐M therapy requires complex target‐specific engineering and carries immune rejection risks^[^ ^11^ ^]^, while our MTM therapy provides a simpler alternative by leveraging macrophages' natural homing to injured myocardium. Through autologous MT, MTM reprograms macrophages into anti‐inflammatory phenotypes without target design, which enhances clinical potential. Unlike CAR‐M's single‐target limitation (e.g., FAP+ fibroblasts)^[^ ^40^ ^]^, MTM exerts multifunctional effects: boosting macrophage migration/phagocytosis and improving cardiomyocyte metabolism via mitochondrial crosstalk. This broader mechanism enables superior post‐MI recovery compared to CAR‐M, positioning MTM as a convenient and promising cardiac repair strategy.^[^ ^41^ ^]^

Benefiting from the robust reparative capacity conferred by MT to macrophages, MTM therapy demonstrates significant efficacy in improving both cardiac structure and function following MI. These findings might also hint potential therapeutic efficacy of MTM for inflammatory diseases associated with macrophage polarization, particularly in conditions such as ischemic stroke and diabetic wounds. In both diabetic wounds and post‐ischemic stroke conditions, macrophages exhibit strikingly similar dysfunctional patterns. The core defect lies in their impaired transition from pro‐inflammatory (M1) to anti‐inflammatory/reparative (M2) phenotypes.^[^ ^42^ ^]^ Consequently, persistent secretion of pro‐inflammatory cytokines (TNF‐α) accompanies insufficient release of reparative factors (TGF‐β, IL‐10) to lead to the adverse outcome.^[^ ^43^ ^]^ Notably, the self‐reinforcing cycle of inflammation‐metabolism dysregulation creates a pathological microenvironment that further locks macrophages in the M1 state.^[^ ^44^, ^45^ ^]^ Our results show that MTM treatment facilitates an accelerated transition of macrophages within the injured area to a reparative phenotype, thereby prolonging the reparative phase and amplifying pro‐healing signaling. Future studies are warranted to prove if such therapeutic strategies targeting polarization of macrophages might also be used for treating other ischemic or inflammatory diseases (e.g., stroke, diabetic wounds).

Looking forward to deciphering the genetic heterogeneity among macrophage populations, the development of a genetically engineered macrophage therapy library will be essential. Coupled with the implementation of standardized protocols for mitochondrial isolation, macrophage engineering, and delivery, such advancements will pave the way for the large‐scale clinical translation of MTM‐based therapies.

## 4 Experimental Section

### Animal Husbandry

The C57BL/6J mice used in this study were sourced from the animal center of the SPF Biotechnology Co., Ltd. (Beijing, China). Male C57BL/6J mice (RRID: IMSR_JAX), aged between 6 and 8 weeks, were housed in ventilated cages within an SPF animal facility, provided with autoclaved normal chow diet, and maintained on a strict 12‐h light/dark cycle. Mice were randomly allocated to different groups. Researchers who performed MI surgery and echocardiography were blinded to mouse treatment. Euthanasia was performed via isoflurane inhalation followed by cervical dislocation. All animal procedures were approved by the Ethical Committee of the Zhongshan Hospital, Fudan University (ZSYY1103) and followed the guidelines set forth in the Guide for the Care and Use of Laboratory Animals (NIH publication 8th Edition, 2011).

### MI Model in Mice

All mice (male and female; 6–8 weeks old; 20–28 g) used for this study were on a C57BL/6J background. Permanent left anterior descending artery (LAD) ligation or a sham operation was performed on experimental animals as described in a previously published protocol.^[^ ^42^ ^]^ Briefly, the mice were anesthetized by inhalation of 2% isoflurane. A small skin cut (1.5 cm) was made over the left chest to expose the heart, and a 6‐0 silk suture was used for permanent ligation of the LAD. Mice that did not survive the first 24 h after the surgery were excluded from analysis. Sham‐operated animals underwent the same procedure without coronary artery ligation. On the third day after MI modeling, mice with EF <50% were selected by echocardiography, with 6–8 mice in each group. All mice were randomly assigned.

### Echocardiography

Cardiac function was assessed by echocardiography performed on a Vevo 2100 Ultrasound system when mice were mildly anesthetized and maintained with 0.5% isoflurane at a heart rate of 500–600 beats min^−1^. Short‐axis M‐mode was used for the measurement of systolic and diastolic ventricular volumes. The measurements on left ventricular fractional shortening (LVFS) and left ventricular ejection fraction (LVEF) were performed using Vevo LAB v5.7.2 from five consecutive cardiac cycles. Left ventricular contractile function was assessed as LVFS.

### Macrophage Isolation and Culturing

Murine bone marrow‐derived macrophages (BMDMs) were isolated and cultured following established protocols. Bone marrow was collected by PBS through the femur, tibia bones of mice using a 1mL syringe needle, and then the bone marrow was treated with red cell lysis buffer (NH 4CL 2009, TBD Science, China). After 5min, an equal amount of PBS was added to stop red cell lysis, shake well for 1min, and then pass through 70 and 40 µm filters respectively. Filtrate was collected and centrifuged (1000r 5min) and then re‐suspended. BMDMs were seeded at a density of 1 × 105 cells mL^−1^ in DMEM culture medium supplemented with 10% FBS, 1% penicillin/streptomycin, and macrophage colony‐stimulating factor (M‐CSF) at a final concentration of 50 ng mL^−1^ (78057, STEMCELL Technologies). The cells were then incubated at 37 °C with 5% CO_2_, and the culture medium was changed at 4 days after isolation. Cells were used within 7–10 days of harvesting.

### Mitochondria Isolation of Cardiomyocytes

Mitochondrial isolation of H9C2 cells was performed using the cell mitochondrial isolation kit (C3601, Beyotime Biotechnology Co., LTD.). H9C2 at logarithmic growth phase (≈90% confluence) were trypsinized (0.25% trypsin, 37 °C, 2 min), followed by centrifugation (300 g, 5 min) and supernatant removal. The cell pellet was homogenized in 8 volumes of cell mitochondrial isolation buffer using thorough mechanical disruption, then sequentially centrifuged (600 g, 10 min, 4 °C) to remove debris, and the resulting supernatant was further centrifuged (11 000 g, 10 min, 4 °C) to obtain the mitochondrial fraction. The final mitochondrial pellet was washed and resuspended in PBS for quantification and subsequent applications.

Mitochondrial isolation of 6–8weeks C57BL/6J mouse hearts was performed using the tissue mitochondrial isolation kit (C3606, Beyotime Biotechnology Co., LTD.). Mice were peritoneally anesthetized with 1% isopentobarbital. After the chest cavity was exposed, the hearts were injected with perfusate until there was almost no blood outflow, then the hearts were completely removed and placed into an EP tube with PBS. Fully cut, natural settlement, wash with PBS. Add 8 times the volume of pre‐cooled special pancreatic enzyme shakes, incubate on ice for 20 min, discard the supernatant, then add 8 volumes of tissue mitochondrial isolation buffer and fully grind. Centrifuge 600 g at 4 °C for 10 min, supernatant was taken and transferred to a new EP tube for further centrifugation (11 000 g,4 °C, 10min). The precipitates obtained, namely the mitochondria from the primary cardiomyocytes, were suspended in PBS and counted for use.

### Mitochondrial Count

The methods for mitochondrial isolation are presented in the Experimental Section. The number of mitochondria isolated using the standard procedure remains relatively consistent. Then the mitochondrial suspension, diluted tenfold, was analyzed three times using a flow cytometer (Beckman Coulter, CytoFLEX). The results in Table S2 showed that the particle concentration in the working suspension was ≈100 particles µL^−1^, which corresponds to a pre‐dilution concentration of 1×10^3^ particles µL^−1^. According to the experimental design in Figure 1A, mitochondrial suspensions with volumes of 10, 20, 40, 80, 160, and 320 µL were added to individual wells of a 6‐well plate, resulting in final mitochondrial quantities of 1×10^4^, 2×10^4^, 4×10^4^, 8×10^4^, 16×10^4^, and 32×10^4^ particles per well, respectively.

### ATP Detection

The ATP determination was carried out using the Beyotime ATP detection kit (S0026). First, lyse the samples in 6‐well plates with 200 µL lysis buffer, mix thoroughly, and centrifuge at 12 000×g for 5 min at 4 °C to collect the supernatant. Meanwhile, prepare a standard curve by diluting ATP standards to seven concentrations (0.01, 0.03, 0.1, 0.3, 1, 3, and 10 µm) using lysis buffer, and keep them on ice. Then, prepare the ATP detection working solution by diluting the assay reagent 1:4 with dilution buffer (100 µL per sample), and store it on ice protected from light. For detection, add 100 µL working solution to each well, incubate at room temperature for 3–5 min to deplete background ATP, followed by adding 20 µL sample or standard, mixing immediately, and measuring RLU (relative light units) with a luminometer after a 2‐s delay. Finally, calculate ATP concentrations using the standard curve (linear range: 0.01–10 µm). Maintain ice‐cold conditions throughout to prevent ATP degradation.

### Efferocytosis Assay

BMDMs or BMDMs treated with mitochondria were cultured in a 6‐well plate and starved overnight (16 h) before the assay. They were both labeled with a red plasma membrane fluorescent probe, Dil (CellTracker CM‐DiI, C7000, Invitrogen, Thermo Fisher Scientific) at 5 µm for 5 min at 37 °C and then for 15 min at 4 °C. Aged RBCs from sheep's blood (Colorado Serum Co., CS31113) were prepared by incubating the cells in PBS (≈25% hematocrit) at 37 °C for 4 days. After washing with PBS, RBCs were further labeled with CFDA‐SE (CFSE) (V12883, Invitrogen, Thermo Fisher Scientific) for 30 min at 37 °C. Subsequently, labeled RBCs were added to the macrophages and incubated for 15 min on ice, followed by 3 h at 37 °C. Images were then captured using the Leica SR5 confocal spinning disk system to visualize the phagocytosis of aged RBCs by BMDMs or BMDMs treated with mitochondria. Phagocytosis efficiency represents the percentage of CFDA+ cells in each group. The phagocytic index represents the CFDA content in positive cells of each group.

### Migration and Invasion Assay of the Transwell System

Use a 24‐well Transwell chamber (4395, Corning HTS Transwell‐24 TC‐treated Reservoir, Sterile) with a diameter of 5 µm. The well‐grown BMDMs were cultured without serum for 24h The control BMDMs and the mitochondrial‐treated BMDMs were seeded in the upper chamber at a density of 1*10^6^ cells mL^−1^. For the invasion assay, the matrix glue should be diluted to 1mg mL^−1^ with serum‐free medium, mixed, and spread 60µL vertically on the chamber, and then incubated at 37 °C for 1–3 h. Wait for the matrix glue to form, and then seed the cells in the upper chamber. Then 500 µL DMEM containing 10% FBS was added to the lower chamber of the 24‐well plate, and the upper chamber was placed in the 24‐well plate with tweezers and incubated at 37 °C and 5%CO_2_ for 24 h. Then, remove the upper chamber, wipe the excess cells in the upper chamber with a cotton swab, add 600µL 4% paraformaldehyde fixing solution, fix the chamber for 30 min, and wash the chamber once with PBS. Then, 600µL crystal violet dyeing solution was added into the clean hole of the 24‐well plate, the upper chamber was put into these holes for 10 min, and wash the chamber with PBS 3 times. Observe under a microscope, take five fields of view, and count with Image J.

### Scratch Assay

The endothelial cells were uniformly seeded into the pore plate (≈5×10⁵ cells per pore) and cultured in a 5% CO_2_ incubator at 37 °C until the cells formed a dense monolayer. Using the bottom of the 200 µL gun head vertical orifice plate, score evenly along the ruler. Rinse gently with PBS to remove shed cells. The supernatant of the BMDMs group and the BMDMs treated with mitochondria group were collected, and a mixture of supernatant and serum‐free medium (the supernatant: serum‐free medium = 1:3) was added into the pore plate. A live cell imager was used to take pictures at 0,6,12, and 24 h. Image J was used to process the images, and the migration distance and the cell mobility of each group were calculated.

(1) $$\mathrm{Cell}\;\mathrm{mobility}\;\left(\%\right)=\left[\left(A_{o}-A_{t}\right)/A_{o}\right]\times 100\%$$

A₀ is the initial area, A_t_ is the t time area.

### Real‐Time PCR

Total RNA from cells or mouse hearts was extracted with TRizol (Thermo Fisher Scientific) according to the manufacturer's instructions. RNA was reverse transcribed into the first‐strand cDNA using the Superscript First‐Strand Synthesis Kit (Invitrogen, Thermo Fisher Scientific). cDNA transcripts were quantified using SYBR Green (BioRad). mRNA levels were normalized to β‐actin and are reported as the fold change over the control.

### Western Blotting

Electrophoresis of proteins was performed using the Tris‐glycine SDS‐PAGE nonreducing system according to the manufacturer's protocol. Briefly, the injured myocardium was lysed in RIPA buffer (Beyotime) with 1× protease and phosphatase inhibitors (Beyotime) and stored at –80 °C until assay. Prior to electrophoresis, whole‐cell lysates were diluted with 5× protein sample buffer (125 mm Tris‐HCl [pH 6.8], 10% SDS, 50% glycerol, 0.06% bromophenol blue, and 1% β‐mercaptoethanol) and incubated at 95 °C for 5 min. Proteins were separated on 12.5% SDS‐PAGE gels and transferred onto a PVDF membrane (Beyotime). Primary antibodies against mouse BAX (2772), Bcl‐2 (15071), and Caspase 3(9662) were from Cell Signaling Technology, and mouse β‐actin (3700) was also from Cell Signaling Technology.

### Mitochondrial Membrane Potential Detection

The mitochondrial membrane potential determination was carried out using the Beyotime TMRE (Tetramethylrhodamine Ethyl Ester) detection kit (C2001S). Take an appropriate amount of TMRE dye (1000X) and dilute it with the detection buffer at a ratio of 1:1000 to obtain the TMRE staining working solution. After incubation at 37 °C for 10 min, aspirate the supernatant and wash twice with preheated cell culture medium. After centrifugation at 1000 rpm for 5 min and resuscitation, fluorescence detection was performed using the PE‐A channel of the flow cytometer.

### ROS Detection

The ROS determination was carried out using the Beyotime DCFH‐DA (2',7'‐Dichlorodihydrofluorescein diacetate) detection kit (S0034S). Take an appropriate amount of DCFH‐DA dye (1000X) and dilute it with PBS at a ratio of 1:1000 to obtain the DCFH‐DA staining working solution. After incubation at 37 °C for 20 min, aspirate the supernatant and wash twice with preheated cell culture medium. After centrifugation at 1000 rpm for 5 min and resuscitation, fluorescence detection was performed using the FITC‐A channel of the flow cytometer.

### Macrophages Polarization Detection

The macrophages polarization determination was carried out using the ABclonal ABflo 450 Rabbit anti‐Mouse CD11b mAb (A26229), ABflo 488 Rat anti‐Mouse F4/80 mAb (A27479), PE Rat anti‐Mouse CD86 mAb (A27137), APC anti‐Mouse CD206 (MMR) mAb (A26785). The antibody was diluted in staining buffer at a ratio of 1:20. First, add the diluted antibody (F4/80, CD11b, CD86), incubate at 4 °C in the dark for 30 min, then centrifuge at 450g at 4 °C for 5 min and discard the supernatant. Fix with cell fixative for 10 min (C1713, Beyotime), centrifuge at 450g for 5 min, and discard the supernatant. Then, permeate with Triton X‐100 for 10 min (C1715, Beyotime), centrifuge at 450g for 5 min, and discard the supernatant. Finally, add CD206 antibody, incubate at 4 °C in the dark for 30 min, centrifuge at 450g for 5 min, and discard the supernatant. Flow cytometry analysis was performed after resuscitation.

### Cytokine and Chemokine Determination

CD206 levels and chemokine CCL2 levels were measured in heart homogenates of injured myocardium and plasma obtained from different groups using ELISA kits according to the manufacturers’ instructions (ABclonal).

### Masson's Trichrome and Collagen Volume Fraction

For paraffin‐embedded sections, heart tissues were fixed in 4% paraformaldehyde (PFA) (DF0131, Leagene Biotechnology), dehydrated using graded alcohols, embedded in paraffin wax, and sectioned at a thickness of 5 µm. After being deparaffinized in xylene, the sections were rehydrated in ethanol and PBS. Use Masson's trichrome to determine collagen volume fraction, and it was calculated as the average of the collagen circumference divided by the LV circumference × 100 in all slices, as described previously.^[^ ^43^ ^]^

### Immunofluorescence Staining

For frozen sections, heart tissues were preserved in OCT compound (BE6104, SAKURA, Japan), rapidly frozen, sectioned at a thickness of 10 µm, and stored at −80 °C. Prior to immunostaining, the samples were fixed with 4% PFA for 10 min at ambient temperature and rinsed with PBS for 10 min. For immunohistological staining, rehydrated sections were treated with sodium citrate buffer for antigen retrieval. The sections were then blocked with QuickBlock Blocking Buffer for Immunol Staining (P0260, Beyotime, China), incubated with primary antibodies overnight at 4 °C, followed by three washes with PBST and a 1‐h incubation with fluorescent secondary antibodies at room temperature. Negative controls were stained with only secondary antibody or IgG isotope antibody to validate antibody specificity and distinguish genuine target staining from the background. Imaging analyses were performed using a Leica SR5 confocal system.

### Mitochondrial Fluorescence Tracing

For in vitro imaging, mitochondria are labeled using MitoTracker Green or Red (Cell Signaling Technology, Danvers, MA, USA) and then co‐incubated with different receptor cells. Confocal microscopy (Thermo Fisher Scientific) was used to image MitoTracker Red‐labeled exogenous mitochondria and MitoTracker Green‐labeled BMDMs endogenous mitochondria. This can show the colocalization of endogenous and exogenous mitochondria, as well as the morphological changes of BMDMs (Figure 1E). To observe the dynamic internalization of transplanted mitochondria, the Lionheart FX Live Cell Imaging Analysis system (BioTek, Winooski, VT, USA) was used to detect MitoTracker Red‐labeled mitochondrial fluorescence intensity within recipient cells (Figure 6I). In vivo imaging, to detect the retention of MitoTracker Green‐labeled mitochondria after transplantation, mouse hearts were imaged with a fluorescence detection system (IVIS Lumina XRMS) at 12h after transplantation (Figure 6A). In addition, MitoTracker Green‐labeled mitochondria in sections of heart tissue were imaged with fluorescence microscopy (Thermo Fisher Scientific) to show their co‐localization with other cells. F4/80 staining represents macrophages in the heart, cTnT staining represents cardiomyocytes, CD31 staining represents endotheliocytes, and α‐SMA staining represents fibroblasts (Figure 6E–G).

### Statistical Analyses

Prism GraphPad 8.0 and SPSS 28.0 were used for the statistical analysis of the experimental data. Significant differences between samples were determined using t‐tests and one‐way ANOVA. Image J software was used for image analysis. Each experiment was conducted in triplicate, and the results are presented as the mean ± standard deviation (mean ± SD).

## Conflict of Interest

The authors declare no conflict of interest.

## Author Contributions

Y.Z. and X.S. contributed equally to this work. Y.Z. and X.S. designed the research and performed most experiments. Y.J. and K.C. developed the experimental methods and completed some key experiments. L.Z., X.G., M.L., and Z.Y. analyzed the data. J.J. provided technical support. Y.Z. and X.S. wrote the manuscript. A.S. and J.G. supervised this work and edited the manuscript.

## Supporting information



Supporting Information

Supplemental Table 1

Supplemental Table 2

## Data Availability

The data that support the findings of this study are available from the corresponding author upon reasonable request.
