# Supplementary material for: Mitochondrial Transplantation Augments the Reparative Capacity of Macrophages Following Myocardial Injury
Source: Adv Sci (Weinh). 2025 Aug 19;12(42):e06337. doi: 10.1002/advs.202506337 (PMC12622452; doi:10.1002/advs.202506337)
Supplement: Supplementary file 1 — Supporting Information [file ADVS-12-e06337-s002.docx]

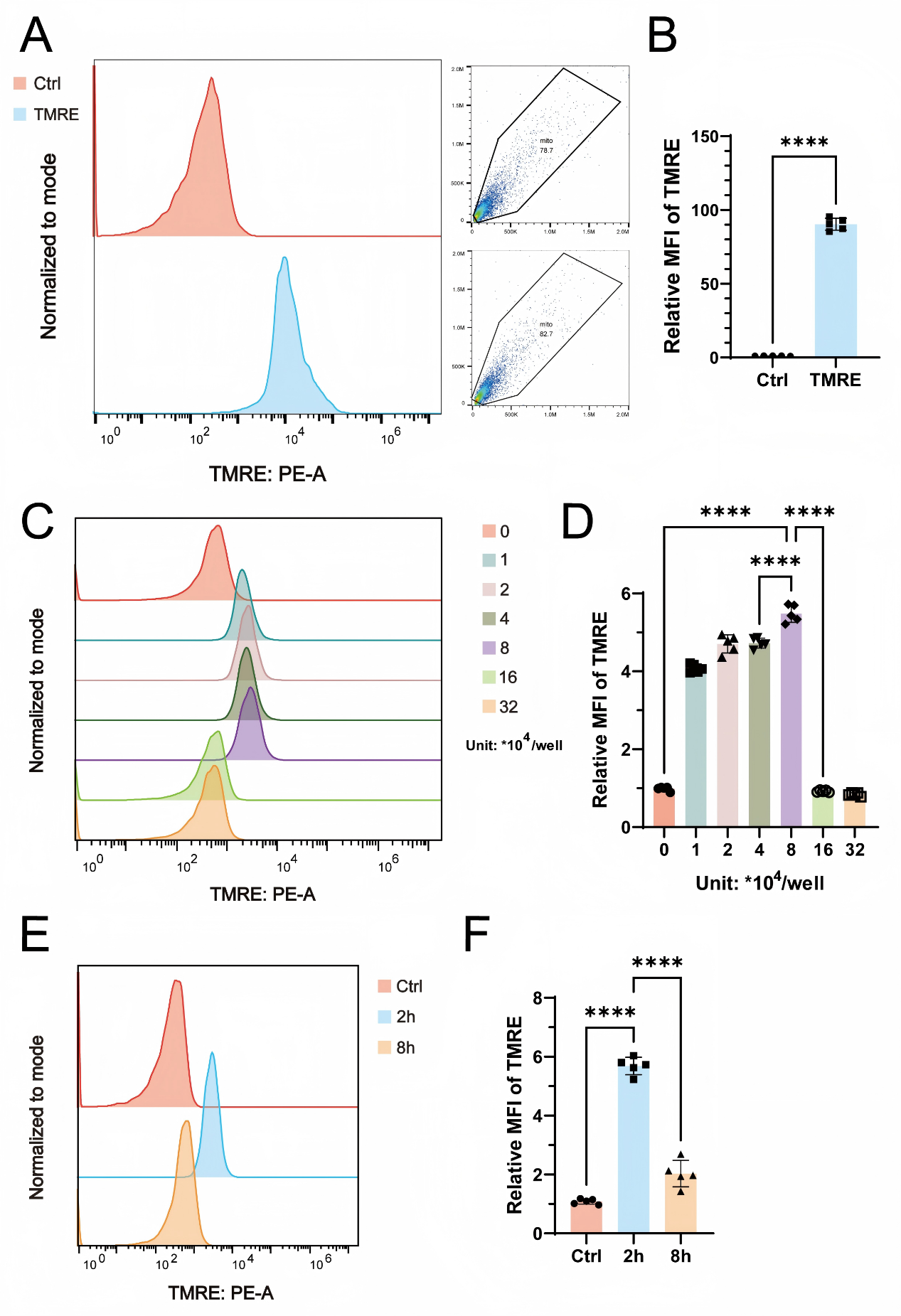


**Supplementary Figure S1. Flow cytometric analysis of varying mitochondrial concentrations and incubation durations on mitochondrial membrane potential in BMDMs.** (A-B): Representative histograms and fluorescence quantitative statistical graphs of mitochondrial membrane potential staining (TMRE) counts of isolated mitochondria (n=5, mean ± SD, t tests). (C-D): Representative histograms and fluorescence quantitative statistical graphs of mitochondrial membrane potential staining (TMRE) counts after co-culture of different concentrations of mitochondrial suspensions (1*10^4^, 2*10^4^, 4*10^4^, 8*10^4^, 16*10^4^, 32*10^4^) with BMDMs for 2 hours respectively (n=5, mean ± SD, one-way ANOVA, Tukey’s multiple comparisons). (E-F): Representative histograms and fluorescence quantitative statistical graphs of mitochondrial membrane potential staining (TMRE) counts after co-culture of the optimal mitochondrial concentration (8*10^4^) with BMDMs for 2 hours and 8 hours (n=5, mean ± SD, one-way ANOVA, Tukey’s multiple comparisons). ****p <0.0001


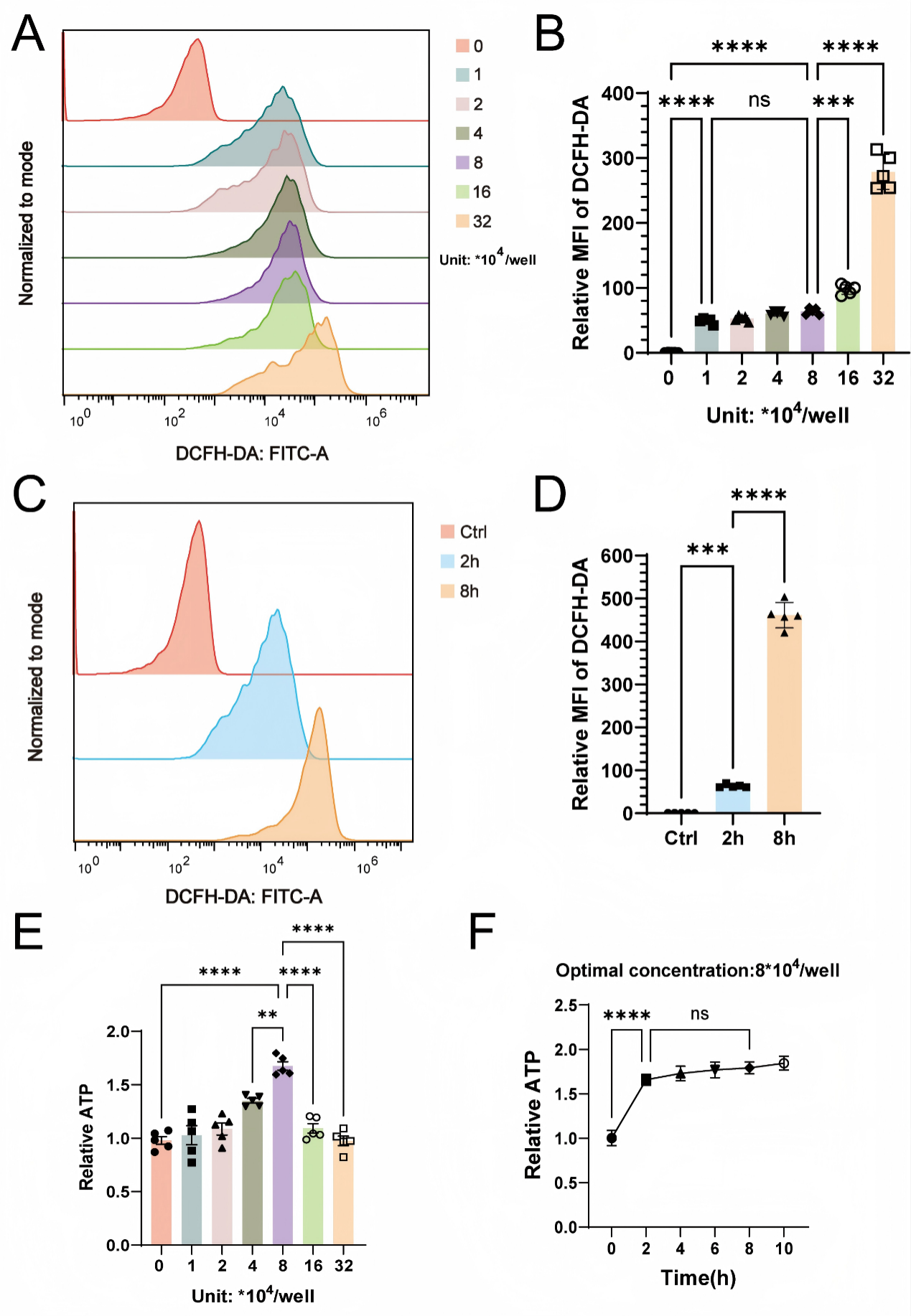


**Supplementary Figure S2. Effects of varying mitochondrial concentrations and incubation durations on ROS and ATP generation in BMDMs.** (A-D): Flow cytometric analysis of ROS generation in BMDMs. (A-B): Representative histograms and fluorescence quantitative statistical graphs of ROS counts after co-culture of different concentrations of mitochondrial suspensions (1*10^4^, 2*10^4^, 4*10^4^, 8*10^4^, 16*10^4^, 32*10^4^) with BMDMs for 2 hours respectively (n=5, mean ± SD, one-way ANOVA, Tukey’s multiple comparisons). (C-D): Representative histograms and fluorescence quantitative statistical graphs of ROS counts after co-culture of the optimal mitochondrial concentration (8*10^4^) with BMDMs for 2 hours and 8 hours (n=5, mean ± SD, one-way ANOVA, Tukey’s multiple comparisons). (E): Histograms of ATP generation after co-culture of different concentrations of mitochondrial suspensions (1*10^4^, 2*10^4^, 4*10^4^, 8*10^4^, 16*10^4^, 32*10^4^) with BMDMs for 2 hours respectively (n=5, mean ± SD, one-way ANOVA, Tukey’s multiple comparisons). (F): Line plot of ATP generation after co-culture of the optimal mitochondrial concentration (8*10^4^) with BMDMs for 0h, 2h, 4h, 6h, 8h, 10h (n=5, mean ± SD, one-way ANOVA, Tukey’s multiple comparisons). **p < 0.01 ***p <0.001 ****p <0.0001


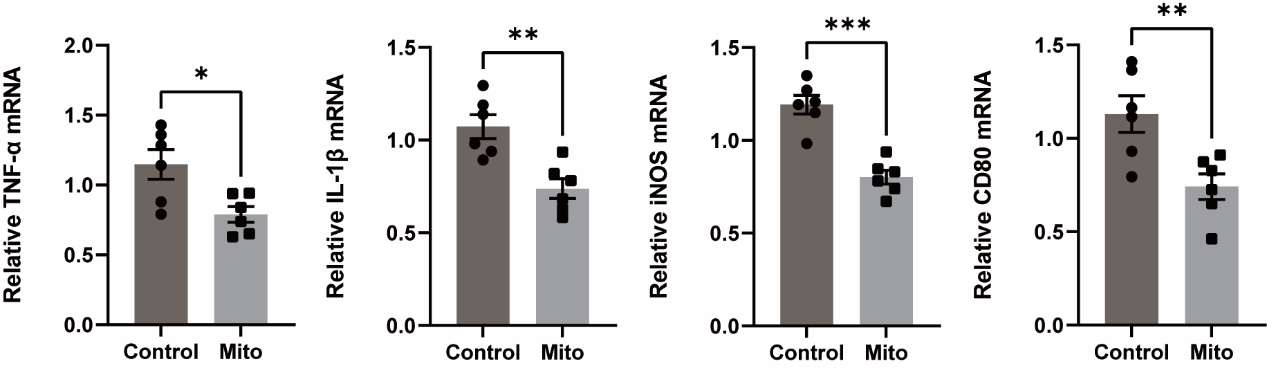


**Supplemental Figure S3. MT decreased the expression of pro-inflammatory cytokines and M1 surface markers of BMDMs.** The mRNA expression of M1 surface markers CD80, iNOS and pro-inflammatory indicators TNF-α, IL-1β in control group and internalized exogenous mitochondria group were detected by q-PCR (n=6, mean ± SD, t tests, *p<0.05 **p < 0.01 ***p <0.001). control=BMDM, mito=BMDM+mito


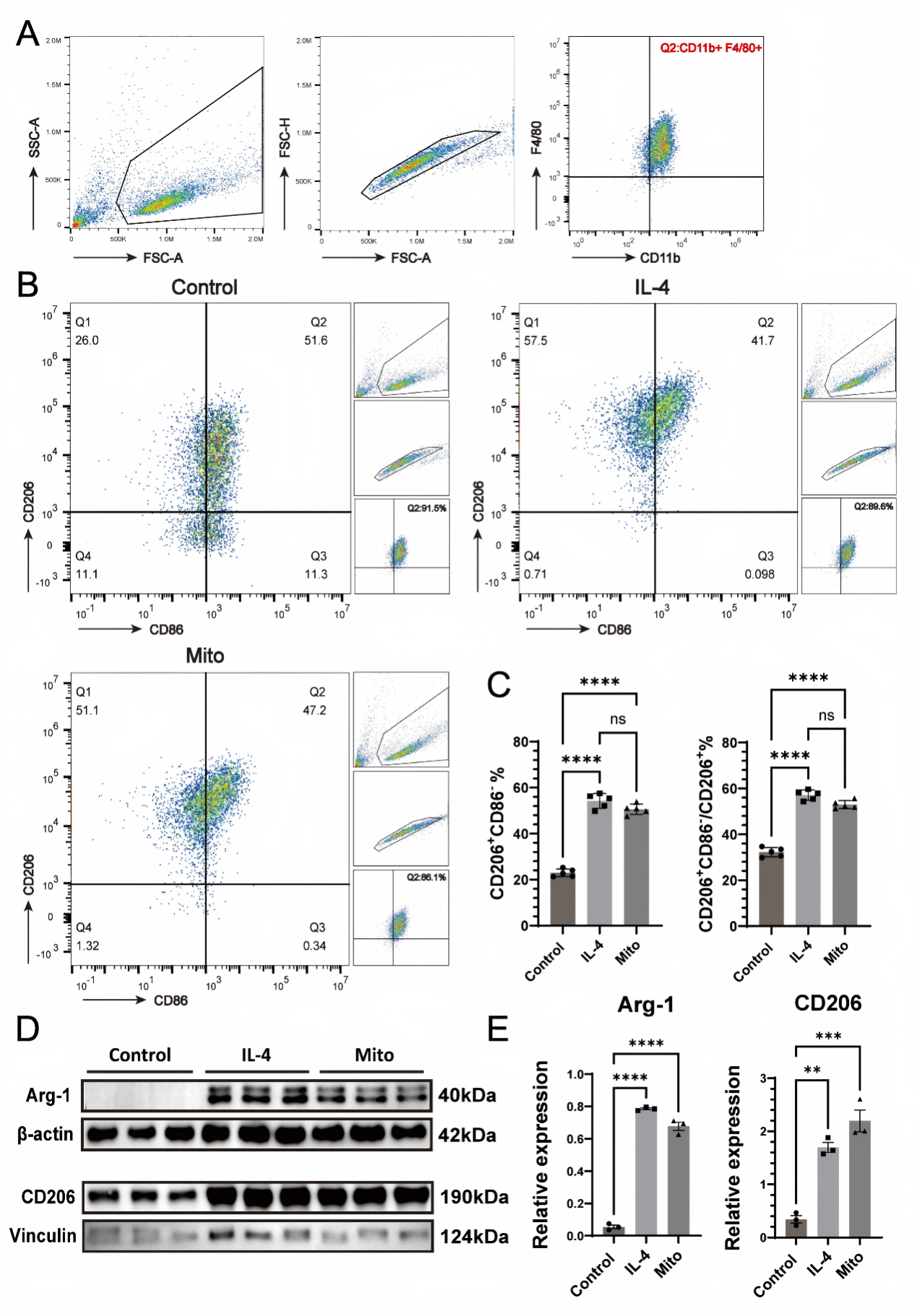


**Supplemental Figure S4. MT promotes the transformation of macrophages to M2 type** (A-C): Flow cytometric analysis of macrophages polarization. (A): The labeling strategy of the macrophage polarization experiment indicates that the CD11b+ F4/80+ cell population in the Q2 quadrant obtained on the far right is the macrophage. (B): Flow cytometry for macrophage polarization analysis in the control group, IL-4 group and Mito group. (C): The proportion of M2 macrophages in each group of Figure S4B. CD206+CD86-% represents the proportion of M2 macrophages, and CD206+CD86-/CD206+% represents the proportion of M2 macrophages among possible repair macrophages. (D): Representative immunoblot images of Arg-1, CD206 proteins from the control, IL-4, Mito group. β-actin and Vinculin were used as internal control. (E): Densitometric quantification of Arg-1, CD206 proteins in each group of Figure S4D (n=3, mean±SEM, one-way ANOVA, Tukey’s multiple comparisons). **p < 0.01 ***p <0.001 ****p <0.0001


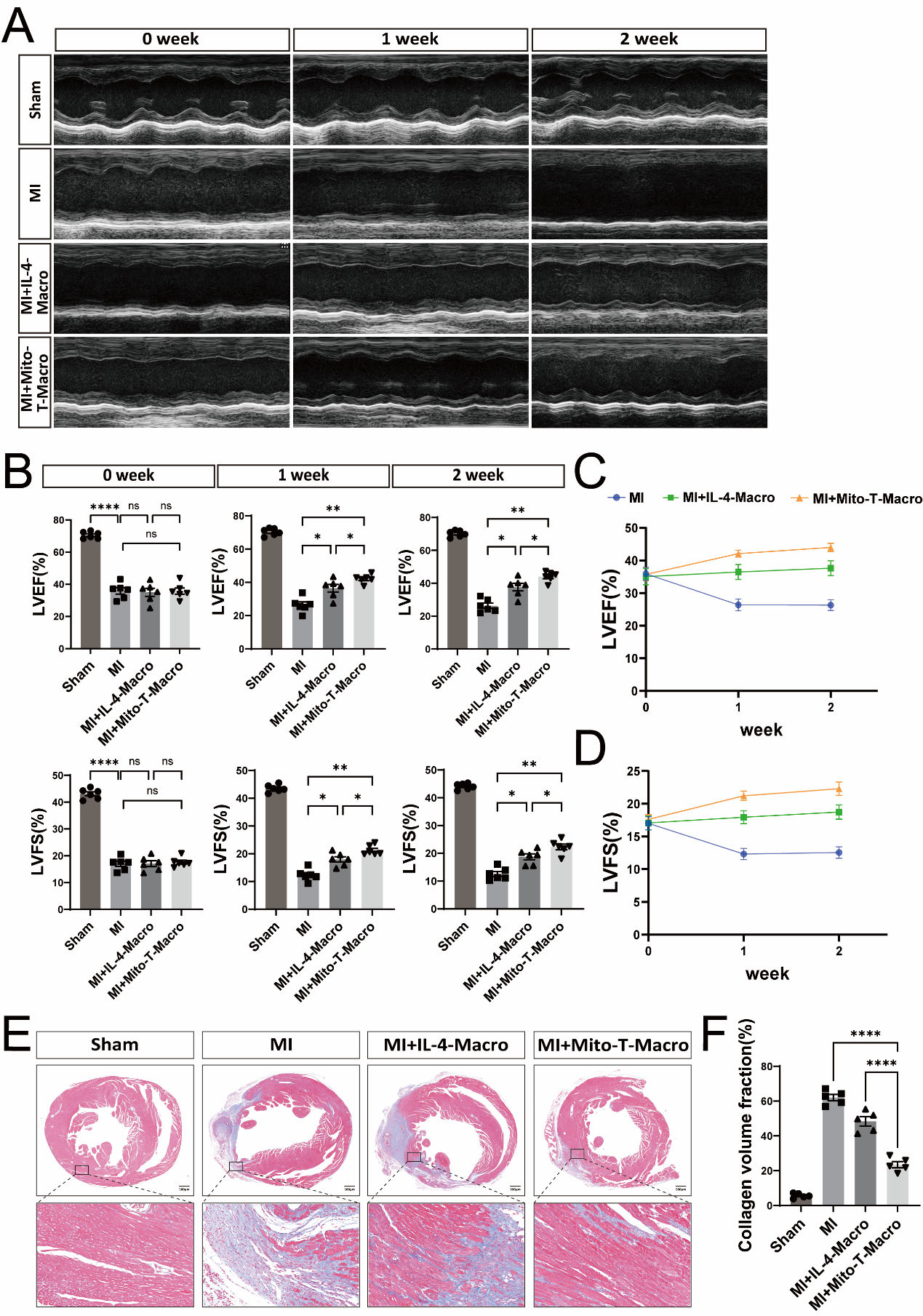


**Supplemental Figure S5. Comparison of cardiac function and fibrosis at 2 weeks post-MI between MTM therapy and IL-4-pretreated macrophages therapy** (A): Representative echocardiographic images (long-axis view) of mice in Sham, MI, MI+IL-4-Macro and MI+Mito-T-Macro groups at 0 week, 1 week and 2 week after MI. (B): Quantitative statistical analysis of left ventricular ejection fraction (LVEF), left ventricular fractional shortening (LVFS) for each group presented in Figure S5A (n=6, mean ± SEM, one-way ANOVA, Tukey’s multiple comparisons). (C-D): Line plots illustrating the changes in LVEF and LVFS of the MI, MI+IL-4-Macro, and MI+Mito-T-Macro groups from 0 week to 2-week post-MI. (E): Representative images of Masson Trichrome staining of mouse hearts in the Sham, MI, MI+IL-4-Macro, and MI+Mito-T-Macro groups at week 2 after MI. Blue signals indicate the fibrotic areas. Scale bar=500 µm. (F): The fibrotic volume proportion in each group of heart tissue specimens in Figure S5E (n=5, mean ± SEM, one-way ANOVA, Tukey’s multiple comparisons). *p < 0.05 **p < 0.01 ***p <0.001 ****p <0.0001


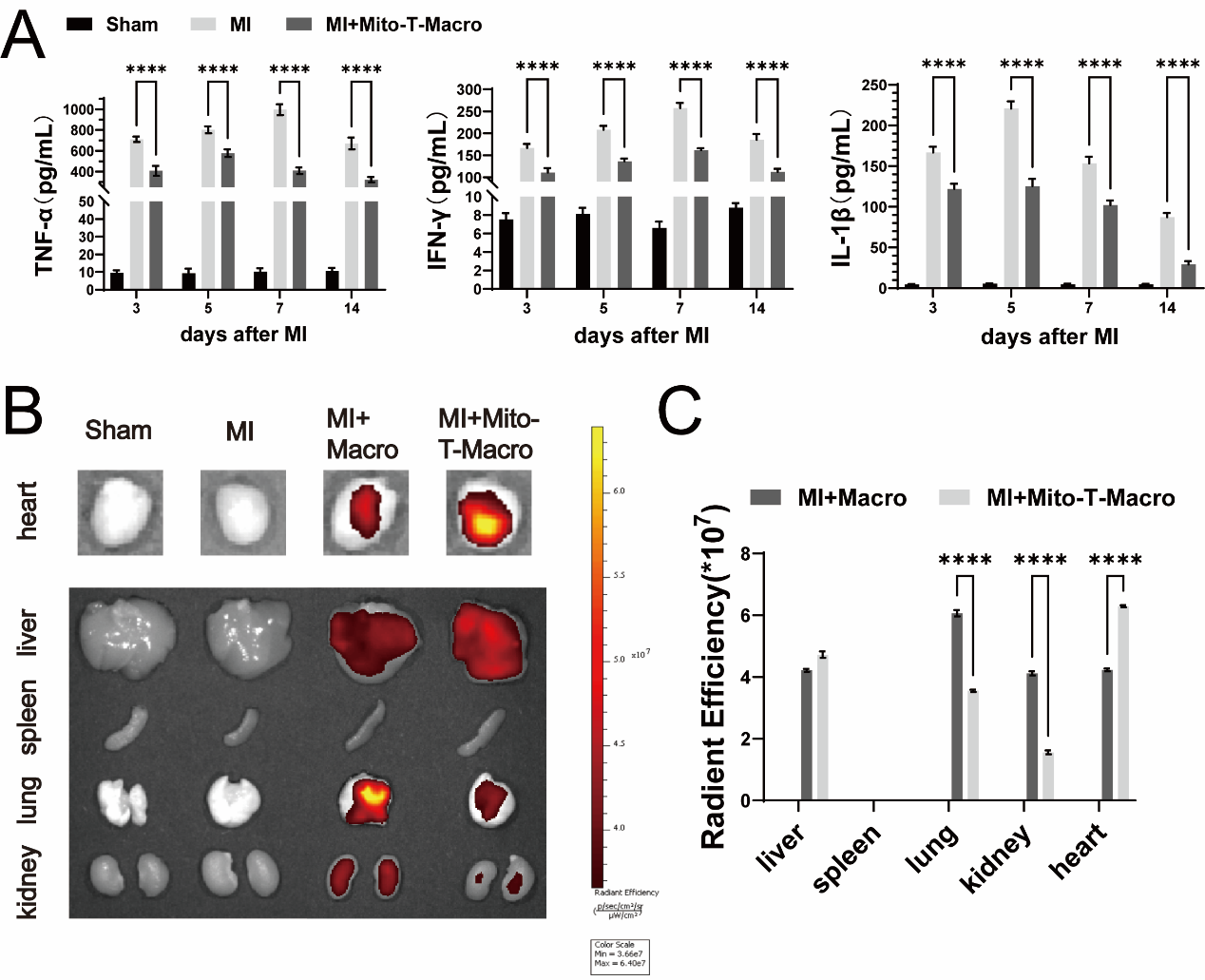


**Supplemental Figure S6. The immune activation profiling and biodistribution of MTM** (A): The concentration of TNF-α, IFN-γ and IL-1β in serum was measured using ELISA on Day 3,5,7,14 for each group post-MI (n=5, mean ± SEM, one-way ANOVA, Tukey’s multiple comparisons, unit: pg/mL). (B): On the third day following MI, mice were administered saline (Sham and MI group), Dil-labeled macrophages (MI+Macro group), and Dil-labeled Mito-T-Macro (MI+Mito-T-Macro group) via tail vein injection. Isolated heart, liver, kidneys, lungs and spleen imaging was conducted 8-12 hours post-injection for all groups. (C): Fluorescence quantitative analysis of *in vivo* heart imaging for each group in Figure S6B (n=5, mean ± SEM, one-way ANOVA, Tukey’s multiple comparisons). ****p <0.0001


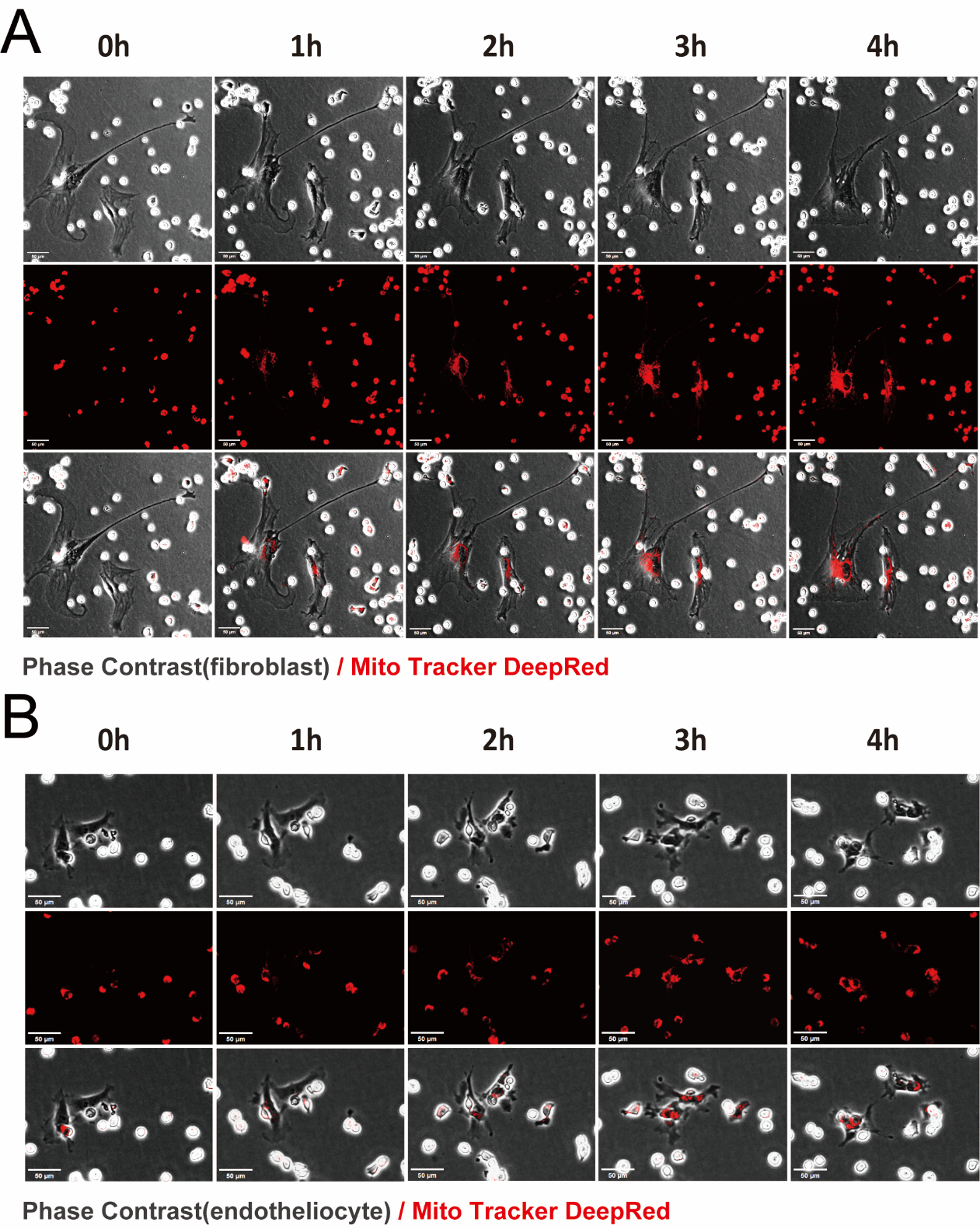


**Supplemental Figure S7. MitoTracker Red-labeled BMDMs cocultured with fibroblasts or endotheliocytes.** Real-time fluorescence imaging of representative living cells captured time-lapse images of mitochondrial-labeled BMDMs co-cultured with fibroblasts or endotheliocytes at 0, 1, 2, 3, and 4 hours.
